# Myosin-X recruits lamellipodin to filopodia tips

**DOI:** 10.1242/jcs.260574

**Published:** 2023-03-02

**Authors:** Ana Popović, Mitro Miihkinen, Sujan Ghimire, Rafael Saup, Max L. B. Grönloh, Neil J. Ball, Benjamin T. Goult, Johanna Ivaska, Guillaume Jacquemet

**Affiliations:** ^1^ Turku Bioscience Centre, University of Turku and Åbo Akademi University, 20520 Turku, Finland; ^2^ Faculty of Science and Engineering, Cell Biology, Åbo Akademi University, 20520 Turku, Finland; ^3^ School of Biosciences, University of Kent, Canterbury, Kent CT2 7NJ, UK; ^4^ Department of Life Technologies, University of Turku, 20520 Turku, Finland; ^5^ InFLAMES Research Flagship Center, University of Turku and Åbo Akademi University, 20520 Turku, Finland; ^6^ Western Finnish Cancer Center (FICAN West), University of Turku, 20520 Turku, Finland; ^7^ Foundation for the Finnish Cancer Institute, Tukholmankatu 8, 00014 Helsinki, Finland; ^8^ Turku Bioimaging, University of Turku and Åbo Akademi University, 20520 Turku, Finland

**Keywords:** Filopodia, MYO10, RAPH1, Cargo transport, Molecular motor

## Abstract

Myosin-X (MYO10), a molecular motor localizing to filopodia, is thought to transport various cargo to filopodia tips, modulating filopodia function. However, only a few MYO10 cargoes have been described. Here, using GFP-Trap and BioID approaches combined with mass spectrometry, we identified lamellipodin (RAPH1) as a novel MYO10 cargo. We report that the FERM domain of MYO10 is required for RAPH1 localization and accumulation at filopodia tips. Previous studies have mapped the RAPH1 interaction domain for adhesome components to its talin-binding and Ras-association domains. Surprisingly, we find that the RAPH1 MYO10-binding site is not within these domains. Instead, it comprises a conserved helix located just after the RAPH1 pleckstrin homology domain with previously unknown functions. Functionally, RAPH1 supports MYO10 filopodia formation and stability but is not required to activate integrins at filopodia tips. Taken together, our data indicate a feed-forward mechanism whereby MYO10 filopodia are positively regulated by MYO10-mediated transport of RAPH1 to the filopodium tip.

## Teaser

**Summary:** Myosin-X transports lamellipodin (RAPH1) to filopodia tips to regulate filopodia functions.

## INTRODUCTION

Cell migration is essential during embryonic development, immune surveillance and wound healing. Misregulation of cell migration is implicated in multiple diseases, including inflammation and cancer. One hallmark of cell motility is a high degree of plasticity, allowing cells to adopt different morphologies and migration modes (Conway and Jacquemet, 2019). A shared feature of efficient cell migration is the ability of cells to probe and interact dynamically with their environments using cellular protrusions, such as filopodia, lamellipodia or pseudopods.

Filopodia are small and dynamic finger-like actin-rich protrusions (1–5 µm in length and 50–200 nm in width) and are often the first point of contact between a cell and its immediate surroundings. Filopodia contain cell surface receptors, such as integrins, cadherins and growth factor receptors, interacting with and interpreting various extracellular cues. Filopodia assembly is primarily driven by the linear polymerization of actin filaments with their barbed ends facing the plasma membrane (Jacquemet et al., 2015). These filaments are further organized into tightly packed bundles by actin-bundling proteins. This unidirectional organization allows molecular motors, such as myosin-X (MYO10), to walk along filopodia and accumulate at their tips (at ∼600 nm/s) (Kerber et al., 2009). By doing so, MYO10 is thought to transport various proteins to filopodia tips, modulating filopodia function (Jacquemet et al., 2015; Arjonen et al., 2014; Berg and Cheney, 2002; Hirano et al., 2011; Zhang et al., 2004). However, only a very few MYO10 cargoes have been proposed to date, with the netrin DCC receptor (Zhu et al., 2007; Wei et al., 2011), integrins (Zhang et al., 2004; Wei et al., 2011) and VASP (Tokuo and Ikebe, 2004) being the principal ones. The MYO10 FERM (protein 4.1R, ezrin, radixin, moesin) domain has been described as the main cargo-binding site in MYO10 (Wei et al., 2011). We previously reported that MYO10 FERM was not required to localize integrins or VASP at filopodia tips. Instead, we found that MYO10 FERM is required for proper integrin activation at filopodia tips (Miihkinen et al., 2021). In addition, we found that deleting the MYO10 FERM domain had little impact on the localization of significant filopodia tip complex components (Miihkinen et al., 2021). These results lead us to question the role of MYO10 as a cargo-transporting molecule.

Here, we set out to identify novel MYO10 cargo molecules. Using GFP-Trap and BioID approaches combined with mass spectrometry, we identified lamellipodin (RAPH1) as a novel MYO10-binding partner. Using structured illumination microscopy, we report that the FERM domain of MYO10 is required for RAPH1 localization and accumulation at filopodia tips; thus, RAPH1 is likely an MYO10 cargo. We map the RAPH1 MYO10-binding site to a previously uninvestigated RAPH1 sequence, and demonstrate that RAPH1 is a critical positive regulator of filopodia formation and stability in cells. Our results indicate that, in filopodia, RAPH1 is not required for integrin activation. Instead, RAPH1 regulates MYO10 filopodia formation and stability.

## RESULTS AND DISCUSSION

### RAPH1 is a putative MYO10 cargo

**Fig. 1.**
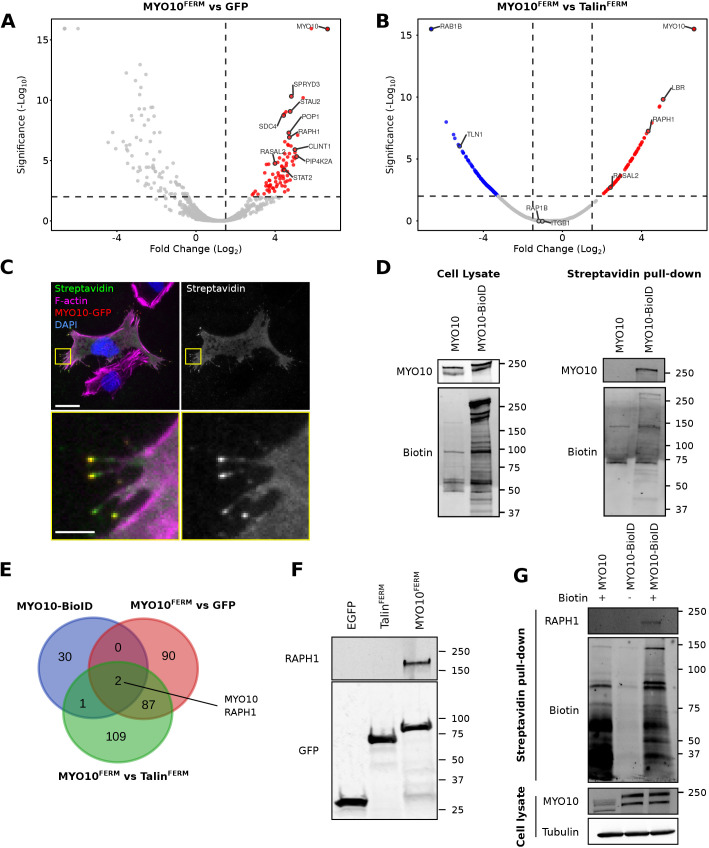
**Mass spectrometry analyses identify RAPH1 as a putative MYO10 binder.** (A,B) Mass spectrometry (MS) analysis of GFP–MYO10^FERM^- and GFP–Talin^FERM^-binding proteins. Comparison of the GFP–MYO10^FERM^ dataset to GFP (A) and GFP–Talin^FERM^ (B) datasets are displayed as volcano plots where the fold-change enrichment is plotted against the significance of the association (see Table S1 for the MS data). The volcano plots were generated using VolcaNoseR (Goedhart and Luijsterburg, 2020). (C) U2-OS cells transiently expressing GFP–MYO10–BioID were plated on fibronectin (FN) in the presence of biotin for 24 h, fixed, stained for biotinylated proteins (using streptavidin), F-actin, and DAPI, and imaged using a spinning disk confocal (SDC) microscope. Scale bars: 25 µm (main); 5 µm (magnification). Note that only one cell in this field of view expresses the GFP–MYO10–BioID construct. Images are representative of three biological repeats. (D) U2-OS cells stably expressing GFP–MYO10–BioID or GFP–MYO10 were plated on FN for 24 h in the presence of biotin. Cells were then lysed, and biotinylated proteins were purified using streptavidin beads. Recruited proteins were analyzed using western blotting and MS (see Table S1 for the MS data). Western blots are displayed (representative of five biological repeats). (E) Venn diagram highlighting the overlap of MYO10-enriched proteins identified from the indicated MS datasets. (F) GFP pulldown in U2-OS cells expressing GFP–MYO10^FERM^, GFP–Talin^FERM^ or GFP alone. RAPH1 recruitment to the bait proteins was then assessed by western blotting (representative of three biological repeats). (G) U2-OS cells stably expressing GFP–MYO10–BioID or GFP-MYO10 were plated on FN for 24 h in the presence or absence of biotin. Cells were then lysed, and biotinylated protein purified using streptavidin beads. RAPH1 biotinylation was then assessed by western blotting (representative of three biological repeats).

To identify novel MYO10 cargo, we searched for proteins that interact specifically with the MyTH4/FERM domain (termed MYO10-FERM here for simplicity), the main cargo-binding site in MYO10 (Wei et al., 2011). We performed GFP pulldowns in U2-OS cells stably expressing GFP, GFP–MYO10^FERM^, or GFP–TLN1^FERM^ (the talin-1 FERM domain), followed by mass spectrometry analysis (Fig. 1A,B). TLN1 FERM was selected as an additional control as it shares structural similarities with the MYO10 FERM domain but performs different functions in cells (Miihkinen et al., 2021). We identified 87 proteins that were specifically enriched in the MYO10-FERM pulldowns (Fig. 1A,B; Table S1). Interestingly, small GTPase regulators, such as RASAL2, ARHGDIA and TRIO, were among the enriched putative MYO10-FERM binders (Table S1).

Next, to narrow the list of putative MYO10 cargo, we tagged GFP–MYO10 with the promiscuous biotin ligase BioID (Roux et al., 2012). BioID was tagged in the C-terminal region, just after the MYO10 FERM domain. In cells, GFP–MYO10–BioID localized to and biotinylated proteins at filopodia tips (Fig. 1C). We purified biotinylated proteins in cells expressing GFP–MYO10 (negative control) or GFP–MYO10–BioID using streptavidin pull-downs (Fig. 1D) and performed mass spectrometry analyses. Somewhat unexpectedly, this approach identified very few proteins, perhaps due to the slow kinetics of the biotin ligase used (Fig. 1E; Table S2). Nevertheless, when comparing our GFP pulldown and BioID datasets, only two proteins, MYO10 itself and lamellipodin (RAPH1), were identified consistently as enriched with MYO10 over controls (Fig. 1E). Western blot analyses confirmed that RAPH1 co-purifies with GFP–MYO10^FERM^ and that RAPH1 is biotinylated in cells expressing GFP–MYO10–BioID (Fig. 1F,G). These results led us to speculate that RAPH1 could be an MYO10 cargo.

### MYO10 FERM is required for RAPH1 localization at filopodia tips

RAPH1 is a member of the Mig-10/RIAM/lamellipodin (MRL) protein family, with MIG-10 being the *Caenorhabditis elegans* ortholog of RAPH1 (Coló et al., 2012). RAPH1 was previously reported to localize to filopodia tips (Krause et al., 2004; Jacquemet et al., 2019), but its contribution to filopodia function remains unknown. Using structured illumination microscopy, we found that RAPH1 specifically accumulates at filopodia tips where it colocalizes with MYO10, whereas RIAM (also known as APBB1IP) is uniformly distributed along filopodia (Fig. S1A). In addition, live-cell imaging at high spatiotemporal resolution indicated that RAPH1 closely follows MYO10 puncta at filopodia tips throughout the filopodia life cycle (Fig. S1B; Movie 1).

**Fig. 2.**
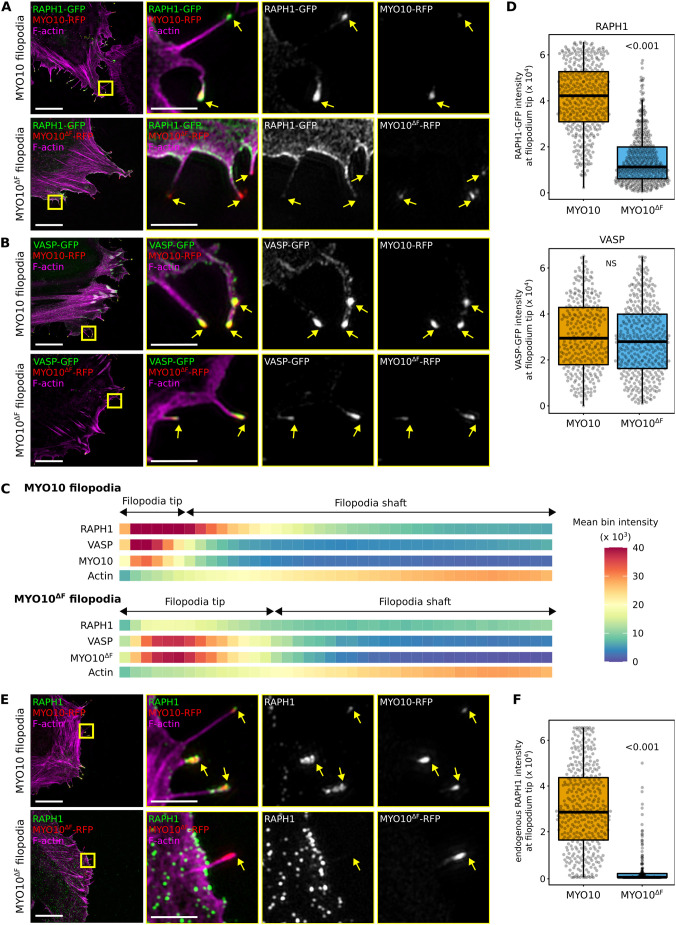
**RAPH1 is recruited to filopodia tips in an MYO10-FERM-dependent manner.** (A–D) U2-OS cells expressing MYO10–RFP or MYO10^ΔF^–RFP together with RAPH1–GFP (A) or VASP–GFP (B) were plated on FN for 2 h, fixed, stained for F-actin and imaged using SIM. (A,B) Representative maximum intensity projection (MIPs) are displayed. Yellow arrows highlight filopodia tips. Scale bars: 20 µm (main); 2 µm (magnifications). (C) Heatmap highlighting the sub-filopodial localization of the proteins imaged in A and B generated from intensity profiles (*n*>300 filopodia per condition; three biological repeats). (D) The average RAPH1 and VASP staining intensity at filopodia tips measured in B are displayed as box plots. (E,F) U2-OS cells expressing MYO10^WT^–RFP or MYO10^ΔF^–RFP were plated on FN for 2 h, fixed, stained for F-actin and endogenous RAPH1, and imaged using SIM. (E) A representative ROI is displayed. Yellow arrows highlight filopodia tips. Scale bars: 2 µm. (F) The average intensity of endogenous RAPH1 at filopodia tips is displayed as box plots (*n*>175 filopodia per condition; three biological repeats). For all panels, the data are shown as dot plots and Tukey boxplots. The whiskers (shown here as vertical lines) extend to data points no further from the box than 1.5× the interquartile range. The *P*-values were determined using a randomization test. NS indicates no statistical difference between the mean values of the highlighted condition and the control.

Our mass spectrometry data indicated that the MYO10 FERM domain binds to RAPH1. Therefore, we investigated the requirement for MYO10-FERM to localize RAPH1 to filopodia tips. We overexpressed an RFP-tagged MYO10 construct lacking the FERM domain (MYO10^ΔF^) in cells (Miihkinen et al., 2021), together with either RAPH1–GFP or VASP–GFP. It was not necessary to suppress the expression of endogenous MYO10 here as MYO10^ΔF^ has a dominant-negative effect in U2-OS cells (Miihkinen et al., 2021). Deleting the MYO10 FERM domain led to a loss of RAPH1 accumulation at filopodia tips (Fig. 2A–D), whereas VASP recruitment remained unaffected (Fig. 2A–D). Importantly, we observe that the accumulation of endogenous RAPH1 was also lost at the tip of MYO10^ΔF^ filopodia (Fig. 2E,F). In line with our results, others reported that the MYO10 FERM domain was required for RAPH1 but not for VASP accumulation in microspikes (Pokrant et al., 2023). Taken together, these findings demonstrate that MYO10 and its FERM domain are required for RAPH1 accumulation at filopodia tips. These results also suggest that, despite containing multiple VASP-binding sites (Krause et al., 2004), RAPH1 is not a prerequisite for VASP localization to MYO10 filopodia.

### RAPH1 directly interacts with MYO10

**Fig. 3.**
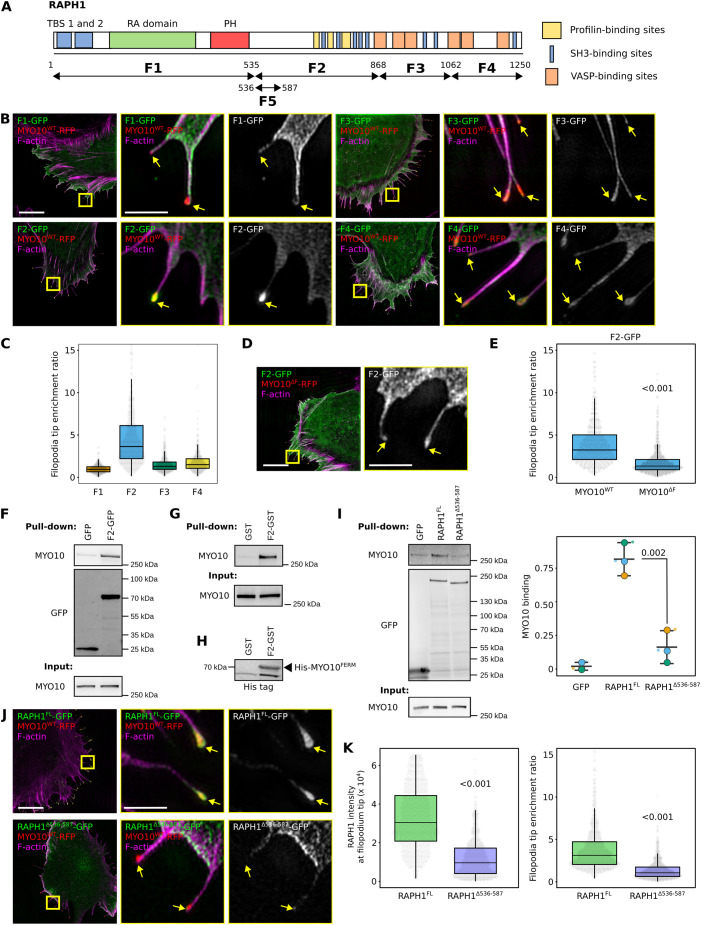
**RAPH1 is recruited to filopodia tips via a region located after its PH domain.** (A) Cartoon representation of RAPH1 domains. The boundaries of the five fragments (F1 to F5) used in this study are highlighted. (B) U2-OS cells expressing MYO10^WT^–RFP and one of the four RAPH1 fragments (F1 to F4) were plated on FN for 2 h, fixed, stained for F-actin and imaged using SIM. Representative maximum intensity projection (MIPs) are displayed. Yellow arrows highlight filopodia tips. Scale bars: 10 µm (main); 2 µm (magnifications). (C) The preferential recruitment of the four RAPH1 fragments to filopodia tips (F1 to F4) was assessed by calculating an enrichment ratio (averaged intensity at filopodia tip versus shaft; >415 filopodia per condition, three biological repeats). (D) U2-OS cells expressing MYO10^WT^–RFP and the GFP–RAPH1^F2^ (GFP-F2) were plated on FN for 2 h, fixed, stained for F-actin, and imaged using SIM. Representative MIPs are displayed. Yellow arrows highlight filopodia tips. Scale bars: 10 µm (main); 2 µm (magnifications). (E) The preferential recruitment of GFP–RAPH1^F2^ to filopodia tips was assessed as in C (>427 filopodia per condition, three biological repeats). (F) GFP pulldowns in MDA-MB-231 cells expressing GFP–RAPH1^F2^ or GFP alone. Endogenous MYO10 recruitment to the bait proteins was then assessed by western blotting (representative of three biological repeats). (G) Pulldowns using recombinant GST–RAPH1^F2^ or GST alone in MDA-MB-231 cell lysates. MYO10 binding to GST–RAPH1^F2^ was then assessed by western blotting (representative of three biological repeats). (H) Pulldowns using recombinant GST–RAPH1^F2^ or GST alone and recombinant His-tagged MYO10^FERM^. MYO10^FERM^ binding to GST–RAPH1^F2^ was then assessed by western blotting (representative of three biological repeats). (I) GFP pulldowns in MDA-MB-231 cells expressing full-length RAPH1–GFP, RAPH1–GFP lacking the F5 fragment (RAPH1^Δ536-587^) or GFP alone. Endogenous MYO10 recruitment to the bait proteins was then assessed by western blotting (representative of three biological repeats). The quantifications of MYO10 recruitment to the bait protein are displayed as a SuperPlot where individual repeats are color-coded (*P*-value calculated using a Welch's *t*-test). Inputs in F, G and I, 5%. (J,K) U2-OS cells expressing MYO10–RFP together with full-length RAPH1–GFP (J) or RAPH1–GFP lacking the F5 fragment (RAPH1^Δ536-587^) were plated on FN for 2 h, fixed, stained for F-actin, and imaged using SIM. (J) Representative MIPs are displayed. Yellow arrows highlight filopodia tips. Scale bars: 10 µm (main); 2 µm (magnifications). (K) The average intensity and preferential recruitment of the two RAPH1 constructs to filopodia tips are displayed as box plots (*n*>800 filopodia per condition; three biological repeats). For all panels, the data are shown as dot plots and Tukey boxplots (except I). The whiskers (shown here as vertical lines) extend to data points no further from the box than 1.5× the interquartile range. The *P*-values were determined using a randomization test (except panel I). NS indicates no statistical difference between the mean values of the highlighted condition and the control.

Next, we sought to identify the MYO10-binding domain(s) within RAPH1. RAPH1 comprises several conserved domains, including a Ras-association (RA) and a pleckstrin homology (PH) domain. RAPH1 also contains known profilin-, VASP- and multiple putative SH3-binding sites (Fig. 3A). Furthermore, previous work has indicated that RAPH1 binds to talin-FERM via two N-terminal talin-binding sites (Lee et al., 2009; Chang et al., 2014) (Fig. 3A; Fig. S2A). As talin-FERM and MYO10-FERM share structural similarities (Miihkinen et al., 2021), we speculated that RAPH1 could bind to MYO10-FERM via these talin-binding sites. To test this hypothesis, we generated a RAPH1 deletion construct lacking both talin-binding sites (Fig. S2B). Deleting both RAPH1 talin-binding sites did not affect RAPH1 localization to filopodia tips indicating that the RAPH1 talin-binding sites are not required for MYO10 interaction (Fig. S2B).

Next, we generated four truncated RAPH1 constructs (named F1 to F4; Fig. 3A) and mapped their filopodia localization (Fig. 3B). Somewhat surprisingly, the RAPH1 fragment F1 containing the PH and the RA domains did not accumulate at filopodia tips. Indeed, among the four constructs tested, only the RAPH1 F2 fragment, which contains the profilin-binding sites, displayed an evident accumulation at filopodia tips (Fig. 3B,C). This required an intact MYO10 FERM domain and was lost in MYO10^ΔF^ filopodia (Fig. 3D,E), indicating that RAPH1 is recruited to filopodia tips via this F2 region.

To validate MYO10–RAPH1 binding, we performed GFP-trap experiments in MDA-MB-231 cells expressing GFP or GFP–RAPH1^F2^. We chose MDA-MB-231 cells for their high endogenous MYO10 protein levels (Jacquemet et al., 2019; 2016). MYO10 co-precipitated with GFP–RAPH1^F2^ (Fig. 3F), validating our microscopy-based assays. In addition, MYO10 was pulled down from cell lysates using recombinant GST–RAPH1^F2^ (Fig. S2C; Fig. 3G), and we detected binding between purified, recombinant GST–RAPH1^F2^ and His-tagged MYO10-FERM proteins (Fig. 3H).

The RAPH1 region encompassed by the RAPH1 F2 fragment is poorly characterized structurally. However, bioinformatic analyses revealed that the amino acid sequence at residues 536–587 is very well conserved (Fig. S3A–E). Expression of a GFP–RAPH1^536-587^ construct (named GFP–RAPH1^F5^) showed that this construct could accumulate at the tip of MYO10-containing filopodia (Fig. S3F). Importantly, the deletion of the amino acid sequence 536 to 587 in full-length RAPH1 was sufficient to nearly abolish RAPH1 binding to MYO10 (Fig. 3I) and block RAPH1 localization and accumulation at filopodia tips (Fig. 3J,K). The subcellular localization of the RAPH1^Δ536-587^ construct appeared otherwise similar to that of full-length RAPH1 and accumulated to the cell leading edge in lamellipodia (Fig. 3J).

Altogether, our data demonstrate a direct interaction between RAPH1 and MYO10, and that RAPH1–MYO10 binding is required for RAPH1 localization to filopodia tips. This interaction is mediated by the MYO10 FERM domain and a previously unexplored, conserved region within RAPH1 located after its PH domain (Fig. 3A).

### RAPH1 modulates filopodia formation and functions

**Fig. 4.**
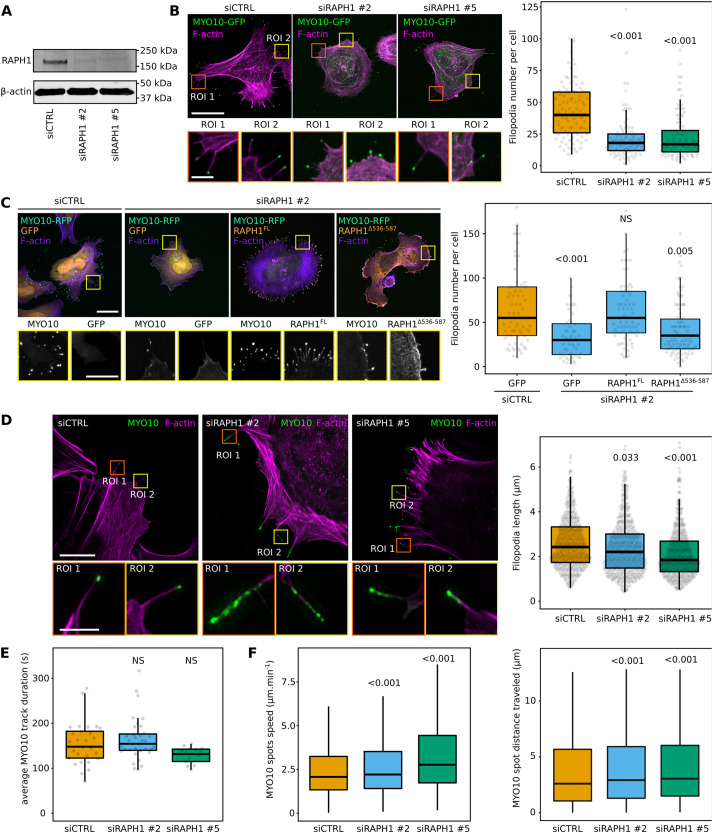
**RAPH1 supports filopodia formation and the stability of the filopodia tip complex.** (A) Efficiency of siRNA-mediated RAPH1 silencing using two different siRNA oligonucleotides in U2-OS cells (representative of three biological repeats). (B) RAPH1-silenced U2-OS cells transiently expressing MYO10–GFP were plated on FN for 2 h, fixed, and the number of MYO10-positive filopodia per cell was quantified (*n*>93 cells per condition, three biological repeats). Scale bars: 25 µm (main); 5 µm (magnifications). (C) RAPH1-silenced U2-OS cells transiently expressing MYO10–RFP together with GFP, RAPH1^FL^–GFP or RAPH1^Δ536-587^–GFP were plated on FN for 2 h, fixed, and the number of MYO10-positive filopodia per cell was quantified (*n*>60 cells per condition, three biological repeats). Scale bars: 25 µm (main); 5 µm (magnifications). (D) RAPH1-silenced U2-OS cells transiently expressing MYO10–GFP were plated on FN, stained for F-actin, and imaged using SIM. Representative maximum intensity projection (MIPs) are displayed. Scale bars: 20 µm (main); 2 µm (magnifications). Quantifications of filopodia length from SIM images are displayed (*n*>530 filopodia per condition; three biological repeats). (E,F) RAPH1-silenced U2-OS cells transiently expressing MYO10–GFP were plated on FN and imaged live using an Airyscan confocal microscope (1 picture every 5 s over 20 min). For each condition, MYO10-positive particles were automatically tracked. (E) The average MYO10 track duration per cell is displayed (three biological repeats, *n*>17 cells per condition). (F) The average speed and the total distance traveled by MYO10 spots are displayed (*n*>9600 filopodia; three biological repeats). For all panels, the data are shown as dot plots (except F) and Tukey boxplots. The whiskers (shown here as vertical lines) extend to data points no further from the box than 1.5× the interquartile range. The *P*-values were determined using a randomization test. NS indicates no statistical difference between the mean values of the highlighted condition and the control.

Next, we investigated the contribution of RAPH1 to filopodia. RAPH1 silencing with two independent siRNA oligonucleotides in U2-OS cells expressing MYO10–GFP significantly reduced MYO10-positive filopodia numbers (Fig. 4A,B). Importantly, this phenotype could be rescued by expressing full-length RAPH1 but not by expressing the RAPH1 construct lacking the F5 fragment (Fig. 4C). In line with these results, others have found that RAPH1 knock-out reduces microspike formation in B16-F1 cells (Pokrant et al., 2023). In addition, we observed that RAPH1 silencing also slightly decreased filopodia length and, interestingly, in a small proportion of RAPH1-silenced cells (below 1%), the filopodia tip complex collapsed, as observed by the dispersed localization of MYO10 along the filopodia shaft (Fig. 4D). Although this phenotype was rare, it was not observed in control cells.

RIAM and RAPH1 have been implicated in modulating integrin activity (Lee et al., 2009). While this role is now well-established for RIAM, it is more controversial for RAPH1 (Coló et al., 2012; Chang et al., 2014; Lafuente et al., 2004; Watanabe et al., 2008). As the MYO10 FERM domain is required to activate integrin at filopodia tips (Miihkinen et al., 2021), we next investigated the role of RAPH1 in modulating integrin activity at filopodia tips (Fig. S4). Using SIM and filopodia mapping analyses, we found that integrin activation at filopodia tip is comparable or slightly elevated in RAPH1-depleted cells compared to that in control cells, indicating that RAPH1 is not required for integrin activation at filopodia tips (Fig. S4).

Finally, we explored the role of RAPH1 in modulating filopodia dynamics in control and RAPH1-silenced U2-OS cells expressing MYO10–GFP. Although the overall filopodia lifetime was unaffected after RAPH1 depletion, MYO10 puncta moved slightly faster and over longer distances than in control cells (Fig. 4E,F), indicating that the filopodia tip complex is more dynamic in RAPH1-depleted cells. The biological significance of these differences remains, however, to be investigated.

### Discussion and conclusions

Here, using two complementary mass spectrometry strategies, we identify RAPH1 as a novel MYO10 interactor. Our results demonstrate that (1) MYO10 is required to target RAPH1 to filopodia tips, and (2) the MYO10–RAPH1 interaction contributes to formation of filopodia containing MYO10. We propose that MYO10 transports RAPH1 to filopodia tips, contributing to filopodia stability via yet unknown mechanisms, possibly involving RAPH1 interactions with other proteins such as VASP. However, our data do not fully exclude the possibility that RAPH1 simply diffuses to filopodia and that MYO10 only contributes to RAPH1 accumulation at filopodia tips without direct transport. Testing this would require performing two-color single-molecule imaging of MYO10 and RAPH1 to see whether these proteins move toward filopodia tips together. However, we find that RAPH1 is not very abundant in filopodia when the MYO10 FERM domain is missing, suggesting that RAPH1 is likely to be actively transported by MYO10.

RAPH1 is presumably in a complex with MYO10, VASP and actin at filopodia tips. In this scenario, MYO10 could tether RAPH1 to filopodia tips using its motor domain, providing resistance against the retrograde actin flow in filopodia (Bornschlögl, 2013; Lidke et al., 2005). Once tethered, RAPH1 would cluster and increase VASP activity by tethering VASP to the actin filaments (Hansen and Mullins, 2015). Although we found that VASP molecules still localize to filopodia tips in the absence of RAPH1, VASP activity will likely be reduced, which could explain the shorter filopodia observed in RAPH1-silenced cells. Therefore, we propose that MYO10-mediated transport of RAPH1 to the filopodium tip is a feed-forward mechanism that positively regulates MYO10 filopodia.

Interestingly, both MYO10 and RAPH1 have been implicated separately as positive regulators of cancer cell migration and invasion in similar contexts (Arjonen et al., 2014; Carmona et al., 2016). In addition, MYO10 and RAPH1 knockout mice share similar phenotypes, such as white belly patches due to defective melanoblast migration (Heimsath et al., 2017; Law et al., 2013). Therefore, it is tempting to speculate that the MYO10–RAPH1 interaction occurring at filopodia tips has strong relevance in health and disease. Future work will investigate the contribution of the MYO10–RAPH1 interaction in regulating cell migration *in vivo*.

## MATERIALS AND METHODS

### Cells

U2-OS osteosarcoma cells and MDA-MB-231 cells were grown in Dulbecco's modified Eagle's medium (DMEM; Sigma, D1152) supplemented with 10% fetal bovine serum (FCS) (Biowest, S1860). U2-OS cells were purchased from DSMZ (Leibniz Institute DSMZ-German Collection of Microorganisms and Cell Cultures, Braunschweig, Germany; ACC 785). MDA-MB-231 cells were provided by ATCC. The U2-OS MYO10-GFP lines were generated by transfecting U2-OS cells using Lipofectamine 3000 (Thermo Fisher Scientific), selected using geneticin (Thermo Fisher Scientific; 400 μg ml^−1^ final concentration), and sorted for green fluorescence using a fluorescence-assisted cell sorter (FACS). All cell lines tested negative for mycoplasma. Cells were not authenticated.

### Antibodies and reagents

Mouse monoclonal antibodies used in this study were anti-β-actin [AC-15, Merck, A1978; dilution for western blotting (WB), 1:1000], anti-His tag (Thermo Fisher Scientific, MA1-21315; dilution for WB, 1:1000), and anti-tubulin (DHSB, clone 12G10; dilution for WB, 1:1000). Rabbit polyclonal antibodies used in this study were anti-RAPH1 [Thermo Fisher Scientific, PA5-110270; dilution for WB, 1:1000; dilution for immunofluorescence (IF) 1:100], anti-MYO10 (Novus Biologicals, 22430002; dilution for WB, 1:1000), and anti-GFP (Abcam, ab290). Biotinylated proteins were detected using Streptavidin conjugated with Alexa Fluor™ 555 (for immunofluorescence, dilution 1:100) or Alexa Fluor™ 647 (for western blots, dilution 1:1000), both provided by Thermo Fisher Scientific (S21381 and S21374). The bovine plasma fibronectin was provided by Merck (341631). DAPI (4',6-diamidino-2-phenylindole, dihydrochloride) was provided by Thermo Fisher Scientific (D1306).

### Plasmids and transfection

U2-OS and MDA-MB-231 cells were transfected using Lipofectamine 3000 and the P3000TM Enhancer Reagent (Thermo Fisher Scientific) according to the manufacturer's instructions.

The EGFPC1-hMyoX (MYO10-GFP) plasmid was Addgene plasmid #47608 (deposited by Emanuel Strehler; Bennett et al., 2007). The mScarlet-MYO10 (MYO10-RFP) construct was described previously (Jacquemet et al., 2019) and is available on Addgene (plasmid #145179). The mScarlet-I-MYO10^ΔF^ (MYO10^ΔF^-RFP) construct was previously described (Miihkinen et al., 2021) and is also available on Addgene (plasmid #145139). The GFP-VASP (mEmerald-VASP-N-10) plasmid was Addgene plasmid #54297 (deposited by Michael Davidson). The GFP-RIAM(1-666) construct was Addgene plasmid #80028 (deposited by Chinten James Lim; Lee et al., 2013). The pcDNA3.1 MCS-BirA(R118G)-HA construct was Addgene plasmid #36047 (deposited by Kyle Roux; Roux et al., 2012). The RAPH1-GFP (EGFP-Lpd) plasmid was kindly provided by Matthias Krause (King's College London, UK).

The GFP–MYO10–BioID construct was generated as follows. Flanking XbaI sites were introduced into BioID by PCR [5′-ATTAGATCTAGAGGATCCAAGGACAACACCGTGCCCCTG-3′, 5′-ATTAGATCTAGACTATGCGTAATCCGGTACATCGTAA-3′, template plasmid: BioID pcDNA3.1 MCS-BirA(R118G)-HA] and the resulting amplicon was then inserted into a unique XbaI site in the EGFPC1-hMyoX plasmid resulting in an EGFP-MYO10-(stop codon)-BioID fusion gene. The stop codon between MYO10 and BioID was then replaced with a codon encoding valine (GTA) using a quick-change mutagenesis kit from Agilent and following the manufacturer's instructions.

The GFP–RAPH1^ΔTBS^ [RAPH1 with amino acids (aa) 2–92 deleted] construct was created by inserting a custom gene block (IDT) in the EGFP-Lpd plasmid using the XhoI/HindIII sites. The sequence of the gene block is provided below:

5′-ATTAGACTCGAGCCGCGATGTGCTCTATAGAGCAGGAGCTCAGCAGCATTGGTTCAGGAAACAGTAAGCGTCAAATCACAGAAACGAAAGCTACTCAGAAATTGCCTGTTAGCCGACATACATTGAAACATGGCACCTTGAAAGGATTATCTTCTTCATCTAATAGGATAGCTAAACCTTCCCATGCCAGCTACTCCTTGGACGACGTCACTGCACAGTTAGAACAGGCCTCTTTGAGTATGGATGAGGCTGCTCAGCAATCTGTACTAGAAGATACTAAACCCTTAGTAACTAATCAGCACAGAAGAACCGCGTCAGCAGGCACAGTGAGTGATGCTGAAGTACACTCTATTAGTAATTCCTCCCATTCCAGCATCACTTCCGCAGCCTCCAGCATGGACTCTTTGGATATTGATAAAGTAACACGCCCTCAAGAGCTGGATTTGACACATCAAGGGCAGCCAATTACTGAGGAAGAACAGGCAGCAAAATTGAAAGCTGAGAAGATCAGAGTTGCCCTAGAGAAAATTAAAGAGGCACAAGTGAAAAAGCTGGTGATCAGAGTCCACATGTCTGATGACAGTTCTAAAACAATGATGGTGGATGAGAGGCAGACAGTAAGACAAGTACTGGATAACCTGATGGACAAATCCCACTGCGGTTATAGTTTAGACTGGTCACTGGTAGAAACCGTTTCTGAATTACAAATGGAGAGAATCTTTGAAGACCATGAAAACTTGGTTGAAAATCTTCTTAATTGGACAAGAGATAGCCAAAACAAGCTTATTAGA-3′.

The RAPH1 fragments F1 (RAPH1 aa 1–535), F2 (RAPH1 aa 535–868), F3 (RAPH1 aa 868–1062), F4 (RAPH1 aa 1062–1250) and F5 (RAPH1 aa 536–587) constructs were purchased from GenScript. Briefly, the gene fragments were synthesized using gene synthesis and cloned into pcDNA3.1(+)-N-eGFP using the BamHI/XhoI sites. The GST–RAPH1^F2^ construct (RAPH1 aa 535–868) was purchased from GenScript. The gene fragment was synthesized using gene synthesis and cloned into pGEX-4T-1 using the BamHI/XhoI sites.

The GFP–RAPH1^Δ536-587^ (RAPH1 aa 536-587 deleted) construct was created by inserting a custom gene block (IDT) in the EGFP-Lpd plasmid using the HindIII/KpnI sites. The sequence of the gene block is provided below:

5′-AAGCTTATATTTATGGAGCGTATAGAAAAATATGCACTTTTCAAAAACCCACAGAATTATCTTTTGGGGAAAAAGGAAACAGCTGAGATGGCAGATAGAAACAAAGAAGTCCTCTTGGAGGAATGTTTTTGTGGAAGTTCTGTAACTGTACCAGAAATTGAAGGAGTCCTTTGGTTGAAGGATGATGGCAAGAAGTCCTGGAAAAAGCGTTATTTTCTCTTGCGAGCATCTGGTATCTACTATGTTCCCAAAGGAAAAGCAAAGGTCTCTCGGGATCTGGTGTGCTTTCTCCAGCTGGATCATGTCAACGTTTATTATGGCCAGGACTATCGGAACAAATACAAAGCACCTACAGACTATTGTCTGGTGCTGAAGCATCCACAAATCCAGAAGAAATCTCAATATATCAAATACCTTTGTTGTGATGATGTGAGGACACTGCATCAGTGGGTCAATGGGATCCGCATTGCAAAGTATGGGAAGCAGCTCTATATGAACTACCAAGAAGCCTTGAAGAGGACAGAGTCAGCCTATGATTGGACTTCCTTATCCAGCTCCAGCATTAAATCGGAAGAGTCCAGCAAGGCCAGAATGGAGTCTATGAATCGGCCCTACACTTCACTTGTGCCCCCTTTATCCCCGCAACCTAAGATAGTCACCCCCTACACTGCTTCACAGCCTTCACCACCTCTACCTCCTCCGCCACCCCCACCTCCTCCTCCACCACCCCCTCCACCACCCCCTCCTCCCCCACTCCCCAGCCAGTCTGCACCTTCTGCAGGCTCAGCAGCCCCAATGTTCGTCAAGTACAGCACAATAACACGGCTACAGAATGCGTCTCAGCATTCAGGGGCCCTGTTTAAGCCGCCAACACCCCCAGTGATGCAGTCACAGTCAGTGAAGCCTCAGATCCTGGTACC-3′.

### siRNA-mediated gene silencing

The expression of RAPH1 was suppressed using 83 nM siRNA and Lipofectamine 3000 (Thermo Fisher Scientific) according to the manufacturer's instructions. siRNAs used were AllStars Negative siRNA control (cat. no. 1027418), RAPH1 siRNA #2 (Hs_RAPH1_2, SI00698642) and RAPH1 siRNA #5 (Hs_RAPH1_5, SI04300982) provided by Qiagen.

### SDS-PAGE and quantitative western blotting

Protein extracts were separated under denaturing conditions by SDS-PAGE and transferred to nitrocellulose membranes using a Mini Blot Module (Invitrogen, B1000). Membranes were blocked for 30 min at room temperature using 1× StartingBlock buffer (Thermo Fisher Scientific, 37578). After blocking, membranes were incubated overnight with the appropriate primary antibody (1:1000 in blocking buffer), washed three times in PBS, and probed for 1 h using a fluorophore-conjugated secondary antibody diluted 1:5000 in the blocking buffer. Membranes were washed three times using PBS over 30 min and scanned using an iBright FL1500 imaging system (Invitrogen).

### GFP-trap pulldown

Cells transiently expressing bait GFP-tagged proteins were lysed in a buffer containing 20 mM HEPES pH 7.4, 75 mM NaCl, 2 mM EDTA, 1% NP-40, as well as a cOmplete™ protease inhibitor tablet (Roche, cat. no. 5056489001), and a phosphatase inhibitor mix (Roche cat. no. 04906837001). Lysates were then centrifuged at 15,000 ***g*** for 5 min at 4°C. Clarified lysates were incubated with GFP-Trap magnetic or agarose beads for 1 h at 4°C. Complexes bound to the beads were isolated by centrifugation, washed three times with ice-cold lysis buffer, and eluted in Laemmli reducing sample buffer for 10 min at 95°C.

### Protein expression and purification

The BL-21(DE3) *Escherichia coli* strain (Merck, cat. no. 70954) was transformed with plasmids encoding the relevant His-tagged or GST-tagged proteins. Bacteria were grown at 37°C in LB medium supplemented with ampicillin (100 µg/ml; Fisher Bioreagents, cat. no. 10419313). Protein expression was induced with IPTG (1 mM; Thermo Fisher Scientific, cat. no. R0392) at 20°C. After 5 h, bacteria were harvested by centrifugation (20 min at 6000 ***g***) and resuspended in resuspension buffer [1× TBS, Pierce Protease Inhibitor Tablet (Thermo Scientific, cat. no. A32963), 1× PMSF (Sigma-Aldrich, cat. no. P7626), 0.05 mg/ml RNase (Roche, cat. no. 10109134001), 0.05 mg/ml DNase (Roche, cat. no. 11284932001)]. Bacteria were then lysed by adding BugBuster (Merck Millipore, cat. no. 70584-4) and a small spoonful of lysozyme (Thermo Scientific, cat. no. 89833). The suspension was mixed at 4°C for 30 min. Cell debris were then pelleted by ultracentrifugation (at 20000 rpm, JA25.50 rotor) at 4°C for 1 h. His-tagged MYO10 FERM was purified using a Protino Ni-TED 2000 packed column (Macherey Nagel, cat. no. 745120.25) according to the manufacturer's instructions. The protein was eluted in multiple 1 ml fractions, supplemented with 1 mM AEBSF (Sigma-Aldrich, cat. no. A8456), and kept at 4°C until needed (for up to 1 week). For GST-tagged proteins, 600 µl of equilibrated glutathione–Sepharose 4B beads (GE Healthcare, cat. no. 17-0756-01) was added to the supernatant and agitated for 1 h at 4°C. Beads were collected and washed four times with TBS supplemented with PMSF (1 mM). Protein-bound beads were stored at −80°C until needed.

### GST pull-down

GST and GST–RAPH1^F2^ Sepharose beads were incubated with 10 mM His-tagged MYO10^FERM^, and the mixture was rotated overnight at 4°C. Beads were then washed four times with TBS supplemented with 1 mM PMSF. Proteins bound to beads were then eluted in 2× Laemmli sample buffer at 80°C. Results were then analyzed by western blotting.

### Proximity biotinylation

U2-OS cells stably expressing GFP–MYO10 or GFP–MYO10–BioID were plated on fibronectin-coated plates in a medium containing 50 μM biotin for 24 h. After washing cells with cold PBS, cells were lysed, and debris were removed by centrifugation (13,000 ***g***, +4°C, 2 min). Biotinylated proteins were then incubated with streptavidin beads (MyOne Streptavidin C1, Invitrogen) for 1 h with rotation at +4°C. Beads were washed twice with 500 μl wash buffer 1 [10% (w/v) SDS], once with 500 μl wash buffer 2 [0.1% (w/v) deoxycholic acid, 1% (w/v) Triton X-100, 1 mM EDTA, 500 mM NaCl, and 50 mM HEPES], and once with 500 μl wash buffer 3 [0.5% (w/v) deoxycholic acid, 0.5% (w/v) NP-40, 1 mM EDTA, and 10 mM Tris/HCl pH 7.4]. Proteins were eluted in 40 μl of 2× reducing sample buffer [100 mM Tris-HCl pH 6.5, 4% (w/v) SDS, 17.5% (v/v) glycerol, 3 mM bromophenol blue, 0.2 M dithiothreitol] for 10 min at 90°C.

### Mass spectrometry analysis

Affinity-captured proteins were separated by SDS-PAGE and allowed to migrate 10 mm into a 4–12% polyacrylamide gel. Following staining with InstantBlue (Expedeon), gel lanes were sliced into five 2-mm bands. The slices were washed using a solution of 50% 100 mM ammonium bicarbonate and 50% acetonitrile until all blue color vanished. Gel slices were washed with 100% acetonitrile for 5–10 min and then rehydrated in a reducing buffer containing 20 mM dithiothreitol in 100 mM ammonium bicarbonate for 30 min at 56°C. Proteins in gel pieces were then alkylated by washing the slices with 100% acetonitrile for 5–10 min and rehydrated using an alkylating buffer of 55 mM iodoacetamide in 100 mM ammonium bicarbonate solution (protected from light, 20 min). Finally, gel pieces were washed with 100% acetonitrile, followed by washes with 100 μl 100 mM ammonium bicarbonate, after which slices were dehydrated using 100% acetonitrile and fully dried using a vacuum centrifuge. Trypsin (0.01 μg/μl; Promega, cat. no. V5111) was used to digest the proteins (37°C overnight). After trypsinization, an equal amount of 100% acetonitrile was added, and gel pieces were further incubated at 37°C for 15 min, followed by peptide extraction using a buffer of 50% acetonitrile and 5% formic acid. The buffer with peptides was collected, and the sample was dried using a vacuum centrifuge. Dried peptides were stored at −20°C. Before liquid chromatography electrospray ionization tandem mass spectrometry (LC-ESI-MS/MS) analysis, dried peptides were dissolved in 0.1% formic acid. The LC-ESI-MS/MS analyses were performed on a nanoflow HPLC system (Easy-nLC1200, Thermo Fisher Scientific) coupled to the Orbitrap Fusion Lumos mass spectrometer (Thermo Fisher Scientific, Bremen, Germany) equipped with a nano-ESI source. Peptides were first loaded on a trapping column and subsequently separated inline on a 15 cm C18 column (75 μm×15 cm, ReproSil-Pur 3 μm 120 Å C18-AQ, Dr Maisch HPLC GmbH, Ammerbuch-Entringen, Germany). The mobile phase consisted of water with 0.1% formic acid (solvent A) and acetonitrile/water [80:20 (v/v)] with 0.1% formic acid (solvent B). Peptides were eluted with 40 min method: from 8% to 43% of solvent B in 30 min, from 43% to 100% solvent B in 2 min, followed by a wash for 8 min at 100% of solvent B. MS data was acquired automatically by using Thermo Xcalibur 4.4 software (Thermo Fisher Scientific). A data-dependent acquisition method consisted of an Orbitrap MS survey scan of mass range 350–1750 *m*/*z* followed by HCD fragmentation for the most intense peptide ions in a full speed mode with a 2.5 s cycle time.

Raw data from the mass spectrometer were submitted to the Mascot search engine using Proteome Discoverer 1.5 (Thermo Fisher Scientific). The search was performed against the human database SwissProt_2021_02, assuming the digestion enzyme trypsin, a maximum of two missed cleavages, an initial mass tolerance of 10 ppm (parts per million) for precursor ions, and a fragment ion mass tolerance of 0.020 Dalton. Cysteine carbamidomethylation was set as a fixed modification, and methionine oxidation was set as a variable modification.

To generate the MYO10–BioID dataset, five biological replicates were combined. Proteins enriched at least twofold in GFP–MYO10–BioID over GFP-MYO10 (based on spectral count) and detected with over five spectral counts (across all repeats) were considered putative MYO10-binding proteins. To generate the MYO10^FERM^ and TLN1^FERM^ datasets, two biological replicates were combined. Proteins enriched at least twofold in MYO10^FERM^ over GFP and over TLN1^FERM^ (based on spectral count) and detected with more than ten spectral counts (across both repeats) were considered putative MYO10-binding proteins. The fold-change enrichment and the significance of the association used to generate the volcano Plots (Fig. 1A,B) were calculated directly in Proteome Discoverer.

### Light microscopy setup

The spinning-disk confocal microscope used was a Marianas spinning-disk imaging system with a Yokogawa CSU-W1 scanning unit on an inverted Zeiss Axio Observer Z1 microscope controlled by SlideBook 6 (Intelligent Imaging Innovations, Inc.). Images were acquired using either an Orca Flash 4 sCMOS camera (chip size 2048×2048; Hamamatsu Photonics) or an Evolve 512 EMCCD camera (chip size 512×512; Photometrics). The objective used was a 100× oil (NA 1.4 oil, Plan-Apochromat, M27) objective.

The structured illumination microscope (SIM) used was DeltaVision OMX v4 (GE Healthcare Life Sciences) fitted with a 60× Plan-Apochromat objective lens, 1.42 NA (immersion oil RI of 1.516) used in SIM illumination mode (five phases×three rotations). Emitted light was collected on a front-illuminated pco.edge sCMOS (pixel size 6.5 mm, readout speed 95 MHz; PCO AG) controlled by SoftWorx.

The confocal microscope used was a laser scanning confocal microscope LSM880 (Zeiss) equipped with an Airyscan detector (Carl Zeiss) and a 40× water (NA 1.2) or 63× oil (NA 1.4) objective. The microscope was controlled using Zen Black (2.3), and the Airyscan was used in standard super-resolution mode.

### Quantification of filopodia numbers and dynamics

For the filopodia formation assays, cells were plated on fibronectin-coated glass-bottom dishes (MatTek Corporation) for 2 h. Samples were fixed for 10 min using a solution of 4% PFA, then permeabilized using a solution of 0.25% (v/v) Triton X-100 for 3 min. Cells were then washed with PBS and quenched using a solution of 1 M glycine for 30 min. Samples were then washed three times in PBS and stored in PBS containing SiR-actin (100 nM; Cytoskeleton; catalog number: CY-SC001) at 4°C until imaging. Just before imaging, samples were washed three times in PBS. Images were acquired using a spinning-disk confocal microscope (100× objective). The number of filopodia per cell was manually scored using Fiji (Schindelin et al., 2012).

To study filopodia stability, U2-OS cells expressing MYO10–GFP were plated on fibronectin for at least 2 h before the start of live imaging (pictures taken every 5 s at 37°C, on an Airyscan microscope, using a 40× objective). All live-cell imaging experiments were performed in normal growth medium, supplemented with 50 mM HEPES, at 37°C and in the presence of 5% CO_2_. Filopodia lifetimes were then measured by identifying and tracking all MYO10 spots using the Fiji plugin TrackMate (Tinevez et al., 2017; Ershov et al., 2022). In TrackMate, the custom Stardist detector and the simple LAP tracker (Linking max distance=1 micron, Gap-closing max distance=0 microns, Gap-closing max frame gap=0 micron) were used. The StarDist 2D model used was trained for 200 epochs on 11 paired image patches [image dimensions: (512, 512), patch size: (512,512)] with a batch size of 2 and a mean absolute error (MAE) loss function, using the StarDist 2D ZeroCostDL4Mic notebook (von Chamier et al., 2021; Schmidt et al., 2018). The training was accelerated using a Tesla K80 GPU.

### Generation of filopodia maps and analysis of filopodia length

U2-OS cells transiently expressing the constructs of interest were plated on high tolerance glass-bottom dishes (MatTek Corporation, coverslip #1.7) pre-coated first with poly-L-lysine (10 µg/ml, 1 h at 37°C; Sigma-Aldrich, cat. no. A-005-M) and then with bovine plasma fibronectin (10 µg/ml, 2 h at 37°C). After 2 h, samples were fixed and permeabilized simultaneously using a solution of 4% (w/v) PFA and 0.25% (v/v) Triton X-100 for 10 min. Cells were then washed with PBS, quenched using a solution of 1 M glycine for 30 min, and, when appropriate, incubated with the primary antibody for 1 h (1:100). After three washes, cells were incubated with a secondary antibody for 1 h (1:100). Samples were then washed three times and incubated with SiR-actin (100 nM in PBS; Cytoskeleton, cat. no. CY- SC001) at 4°C until imaging (minimum length of staining, overnight at 4°C; maximum length, 1 week). Just before imaging, samples were washed three times in PBS and mounted in Vectashield (Vector Laboratories).

To map the localization of each protein within filopodia, images were first processed in Fiji (Schindelin et al., 2012), and data were analyzed using R software as previously described (Jacquemet et al., 2019). Briefly, in Fiji, the brightness and contrast of each image were automatically adjusted using, as an upper maximum, the brightest cellular structure labeled in the field of view. In Fiji, line intensity profiles (1-pixel width) were manually drawn from filopodia tip to base (defined by the intersection of the filopodia and the lamellipodium). The intensity profile lines were drawn from a merged image to avoid any bias in the analysis. All visible filopodia in each image were analyzed and exported for further analysis (export was performed using the ‘Multi Plot’ function). For each staining, line intensity profiles were then compiled and analyzed in R software. To homogenize filopodia length, each line intensity profile was binned into 40 bins (using the median value of pixels in each bin and the R function ‘tapply’). The map of each protein of interest was created by averaging hundreds of binned intensity profiles. The length of each filopodium analyzed was directly extracted from the line intensity profiles.

The preferential recruitment of protein to filopodia tips or shafts was assessed by calculating an enrichment ratio where the averaged intensity of the signal at the filopodia tip (bin 1–6) was divided by the averaged intensity at the filopodia shaft (bin 7–40).

### Quantification and statistical analysis

Randomization tests were performed using the online tool PlotsOfDifferences (https://huygens.science.uva.nl/PlotsOfDifferences/) (Goedhart, 2019 preprint). Dot plots were generated using PlotsOfData (Postma and Goedhart, 2019). Volcano Plots were generated using VolcaNoseR (Goedhart and Luijsterburg, 2020). Superplots were generated using SuperPlotsOfData (Goedhart, 2021). All numerical raw data and associated statistical analyses (including effect size) are available in Table S2. The ImageJ macro and the R code used to generate the filopodia maps were previously described and are available on GitHub (https://github.com/guijacquemet/FiloMAP).

## Supplementary Material

10.1242/joces.260574_sup1 Supplementary information

## References

[JCS260574C1] Arjonen, A., Kaukonen, R., Mattila, E., Rouhi, P., Högnäs, G., Sihto, H., Miller, B. W., Morton, J. P., Bucher, E., Taimen, P. et al. (2014). Mutant p53-associated myosin-X upregulation promotes breast cancer invasion and metastasis. *J. Clin. Invest.* 124, 1069-1082. 10.1172/JCI67280 24487586 PMC3934176

[JCS260574C2] Bennett, R. D., Mauer, A. S. and Strehler, E. E. (2007). Calmodulin-like protein increases filopodia-dependent cell motility via up-regulation of myosin-10. *J. Biol. Chem.* 282, 3205-3212. 10.1074/jbc.M607174200 17130134

[JCS260574C3] Berg, J. S. and Cheney, R. E. (2002). Myosin-X is an unconventional myosin that undergoes intrafilopodial motility. *Nat. Cell Biol.* 4, 246-250. 10.1038/ncb762 11854753

[JCS260574C4] Bornschlögl, T. (2013). How filopodia pull: What we know about the mechanics and dynamics of filopodia. *Cytoskeleton* 70, 590-603. 10.1002/cm.21130 23959922

[JCS260574C5] Carmona, G., Perera, U., Gillett, C., Naba, A., Law, A.-L., Sharma, V. P., Wang, J., Wyckoff, J., Balsamo, M., Mosis, F. et al. (2016). Lamellipodin promotes invasive 3D cancer cell migration via regulated interactions with Ena/VASP and SCAR/WAVE. *Oncogene* 35, 5155-5169. 10.1038/onc.2016.47 26996666 PMC5031503

[JCS260574C6] Chang, Y.-C., Zhang, H., Franco-Barraza, J., Brennan, M. L., Patel, T., Cukierman, E. and Wu, J. (2014). Structural and mechanistic insights into the recruitment of Talin by RIAM in integrin signaling. *Structure* 22, 1810-1820. 10.1016/j.str.2014.09.020 25465129 PMC4255149

[JCS260574C7] Coló, G. P., Lafuente, E. M. and Teixidó, J. (2012). The MRL proteins: Adapting cell adhesion, migration and growth. *Eur. J. Cell Biol.* 91, 861-868. 10.1016/j.ejcb.2012.03.001 22555291

[JCS260574C8] Conway, J. R. W. and Jacquemet, G. (2019). Cell matrix adhesion in cell migration. *Essays Biochem.* 63, 535-551. 10.1042/EBC20190012 31444228

[JCS260574C9] Ershov, D., Phan, M.-S., Pylvänäinen, J. W., Rigaud, S. U., Le Blanc, L., Charles-Orszag, A., Conway, J. R. W., Laine, R. F., Roy, N. H., Bonazzi, D. et al. (2022). TrackMate 7: integrating state-of-the-art segmentation algorithms into tracking pipelines. *Nat. Methods* 19, 829-832. 10.1038/s41592-022-01507-1 35654950

[JCS260574C10] Goedhart, J. (2019). PlotsOfDifferences – a web app for the quantitative comparison of unpaired data. *bioRxiv* 578575. 10.1101/578575

[JCS260574C11] Goedhart, J. (2021). SuperPlotsOfData-a web app for the transparent display and quantitative comparison of continuous data from different conditions. *Mol. Biol. Cell* 32, 470-474. 10.1091/mbc.E20-09-0583 33476183 PMC8101441

[JCS260574C12] Goedhart, J. and Luijsterburg, M. S. (2020). VolcaNoseR is a web app for creating, exploring, labeling and sharing volcano plots. *Sci. Rep.* 10, 20560. 10.1038/s41598-020-76603-3 33239692 PMC7689420

[JCS260574C13] Hansen, S. D. and Mullins, R. D. (2015). Lamellipodin promotes actin assembly by clustering Ena/VASP proteins and tethering them to actin filaments. *eLife* 4, e06585. 10.7554/eLife.06585 26295568 PMC4543927

[JCS260574C14] Heimsath, E. G., Yim, Y.-I., Mustapha, M., Hammer, J. A. and Cheney, R. E. (2017). Myosin-X knockout is semi-lethal and demonstrates that myosin-X functions in neural tube closure, pigmentation, hyaloid vasculature regression, and filopodia formation. *Sci. Rep.* 7, 17354. 10.1038/s41598-017-17638-x 29229982 PMC5725431

[JCS260574C15] Hirano, Y., Hatano, T., Takahashi, A., Toriyama, M., Inagaki, N. and Hakoshima, T. (2011). Structural basis of cargo recognition by the myosin-X MyTH4-FERM domain. *EMBO J.* 30, 2734-2747. 10.1038/emboj.2011.177 21642953 PMC3155308

[JCS260574C16] Jacquemet, G., Hamidi, H. and Ivaska, J. (2015). Filopodia in cell adhesion, 3D migration and cancer cell invasion. *Curr. Opin. Cell Biol.* 36, 23-31. 10.1016/j.ceb.2015.06.007 26186729

[JCS260574C17] Jacquemet, G., Baghirov, H., Georgiadou, M., Sihto, H., Peuhu, E., Cettour-Janet, P., He, T., Perälä, M., Kronqvist, P., Joensuu, H. et al. (2016). L-type calcium channels regulate filopodia stability and cancer cell invasion downstream of integrin signalling. *Nat. Commun.* 7, 13297. 10.1038/ncomms13297 27910855 PMC5146291

[JCS260574C18] Jacquemet, G., Stubb, A., Saup, R., Miihkinen, M., Kremneva, E., Hamidi, H. and Ivaska, J. (2019). Filopodome mapping identifies p130Cas as a mechanosensitive regulator of Filopodia stability. *Curr. Biol* 29, 202-216.e7. 10.1016/j.cub.2018.11.053 30639111 PMC6345628

[JCS260574C19] Kerber, M. L., Jacobs, D. T., Campagnola, L., Dunn, B. D., Yin, T., Sousa, A. D., Quintero, O. A. and Cheney, R. E. (2009). A novel form of motility in filopodia revealed by imaging myosin-X at the single-molecule level. *Curr. Biol.* 19, 967-973. 10.1016/j.cub.2009.03.067 19398338 PMC2817954

[JCS260574C20] Krause, M., Leslie, J. D., Stewart, M., Lafuente, E. M., Valderrama, F., Jagannathan, R., Strasser, G. A., Rubinson, D. A., Liu, H., Way, M. et al. (2004). Lamellipodin, an Ena/VASP ligand, is implicated in the regulation of lamellipodial dynamics. *Dev. Cell* 7, 571-583. 10.1016/j.devcel.2004.07.024 15469845

[JCS260574C21] Lafuente, E. M., van Puijenbroek, A. A. F. L., Krause, M., Carman, C. V., Freeman, G. J., Berezovskaya, A., Constantine, E., Springer, T. A., Gertler, F. B. and Boussiotis, V. A. (2004). RIAM, an Ena/VASP and profilin ligand, interacts with Rap1–GTP and mediates Rap1-induced adhesion. *Dev. Cell.* 7, 585-595. 10.1016/j.devcel.2004.07.021 15469846

[JCS260574C22] Law, A.-L., Vehlow, A., Kotini, M., Dodgson, L., Soong, D., Theveneau, E., Bodo, C., Taylor, E., Navarro, C., Perera, U. et al. (2013). Lamellipodin and the Scar/WAVE complex cooperate to promote cell migration in vivo. *J. Cell Biol.* 203, 673-689. 10.1083/jcb.201304051 24247431 PMC3840943

[JCS260574C23] Lee, H.-S., Lim, C. J., Puzon-McLaughlin, W., Shattil, S. J. and Ginsberg, M. H. (2009). RIAM activates integrins by linking talin to ras GTPase membrane-targeting sequences. *J. Biol. Chem.* 284, 5119-5127. 10.1074/jbc.M807117200 19098287 PMC2643525

[JCS260574C24] Lee, H.-S., Anekal, P., Lim, C. J., Liu, C.-C. and Ginsberg, M. H. (2013). Two modes of integrin activation form a binary molecular switch in adhesion maturation. *Mol. Biol. Cell* 24, 1354-1362. 10.1091/mbc.E12-09-0695 23468527 PMC3639047

[JCS260574C25] Lidke, D. S., Lidke, K. A., Rieger, B., Jovin, T. M. and Arndt-Jovin, D. J. (2005). Reaching out for signals. *J. Cell Biol.* 170, 619-626. 10.1083/jcb.200503140 16103229 PMC2171515

[JCS260574C26] Miihkinen, M., Grönloh, M. L. B., Popović, A., Vihinen, H., Jokitalo, E., Goult, B. T., Ivaska, J. and Jacquemet, G. (2021). Myosin-X and talin modulate integrin activity at filopodia tips. *Cell Rep.* 36, 109716. 10.1016/j.celrep.2021.109716 34525374 PMC8456781

[JCS260574C27] Pokrant, T., Hein, J. I., Körber, S., Disanza, A., Pich, A., Scita, G., Rottner, K. and Faix, J. (2023). Ena/VASP clustering at microspike tips involves lamellipodin but not I-BAR proteins, and absolutely requires unconventional myosin-X. *Proc. Natl. Acad. Sci. USA* 120, e2217437120. 10.1073/pnas.2217437120 36598940 PMC9926217

[JCS260574C28] Postma, M. and Goedhart, J. (2019). PlotsOfData—A web app for visualizing data together with their summaries. *PLoS Biol.* 17, e3000202. 10.1371/journal.pbio.3000202 30917112 PMC6453475

[JCS260574C29] Roux, K. J., Kim, D. I., Raida, M. and Burke, B. (2012). A promiscuous biotin ligase fusion protein identifies proximal and interacting proteins in mammalian cells. *J. Cell Biol.* 196, 801-810. 10.1083/jcb.201112098 22412018 PMC3308701

[JCS260574C30] Schindelin, J., Arganda-Carreras, I., Frise, E., Kaynig, V., Longair, M., Pietzsch, T., Preibisch, S., Rueden, C., Saalfeld, S., Schmid, B. et al. (2012). Fiji: an open-source platform for biological-image analysis. *Nat. Methods* 9, 676-682. 10.1038/nmeth.2019 22743772 PMC3855844

[JCS260574C31] Schmidt, U., Weigert, M., Broaddus, C. and Myers, G. (2018). Cell detection with star-convex polygons. In *Medical Image Computing and Computer Assisted Intervention – MICCAI 2018* (ed. A.F. Frangi, J.A. Schnabel, C. Davatzikos, C. Alberola-López and G. Fichtinger), pp. 265-273. Cham: Springer International Publishing. 10.1007/978-3-030-00934-2_30

[JCS260574C32] Tinevez, J.-Y., Perry, N., Schindelin, J., Hoopes, G. M., Reynolds, G. D., Laplantine, E., Bednarek, S. Y., Shorte, S. L. and Eliceiri, K. W. (2017). TrackMate: an open and extensible platform for single-particle tracking. *Methods* 115, 80-90. 10.1016/j.ymeth.2016.09.016 27713081

[JCS260574C33] Tokuo, H. and Ikebe, M. (2004). Myosin X transports Mena/VASP to the tip of filopodia. *Biochem. Biophys. Res. Commun.* 319, 214-220. 10.1016/j.bbrc.2004.04.167 15158464

[JCS260574C34] von Chamier, L., Laine, R. F., Jukkala, J., Spahn, C., Krentzel, D., Nehme, E., Lerche, M., Hernández-Pérez, S., Mattila, P. K., Karinou, E. et al. (2021). Democratising deep learning for microscopy with ZeroCostDL4Mic. *Nat. Commun* 12, 2276. 10.1038/s41467-021-22518-0 33859193 PMC8050272

[JCS260574C35] Watanabe, N., Bodin, L., Pandey, M., Krause, M., Coughlin, S., Boussiotis, V. A., Ginsberg, M. H. and Shattil, S. J. (2008). Mechanisms and consequences of agonist-induced talin recruitment to platelet integrin αIIbβ3. *J. Cell Biol.* 181, 1211-1222. 10.1083/jcb.200803094 18573917 PMC2442211

[JCS260574C36] Wei, Z., Yan, J., Lu, Q., Pan, L. and Zhang, M. (2011). Cargo recognition mechanism of myosin X revealed by the structure of its tail MyTH4-FERM tandem in complex with the DCC P3 domain. *Proc. Natl. Acad. Sci. U. S. A* 108, 3572-3577. 10.1073/pnas.1016567108 21321230 PMC3048157

[JCS260574C37] Zhang, H., Berg, J. S., Li, Z., Wang, Y., Lång, P., Sousa, A. D., Bhaskar, A., Cheney, R. E. and Strömblad, S. (2004). Myosin-X provides a motor-based link between integrins and the cytoskeleton. *Nat. Cell Biol.* 6, 523-531. 10.1038/ncb1136 15156152

[JCS260574C38] Zhu, X.-J., Wang, C.-Z., Dai, P.-G., Xie, Y., Song, N.-N., Liu, Y., Du, Q.-S., Mei, L., Ding, Y.-Q. and Xiong, W.-C. (2007). Myosin X regulates netrin receptors and functions in axonal path-finding. *Nat. Cell Biol.* 9, 184-192. 10.1038/ncb1535 17237772

