# Supplementary material for: Myosin-X recruits lamellipodin to filopodia tips
Source: J Cell Sci. 2023 Mar 2;136(5):jcs260574. doi: 10.1242/jcs.260574 (PMC10022686; doi:10.1242/jcs.260574)
Supplement: Supplementary information [file joces-136-260574-s1.pdf]

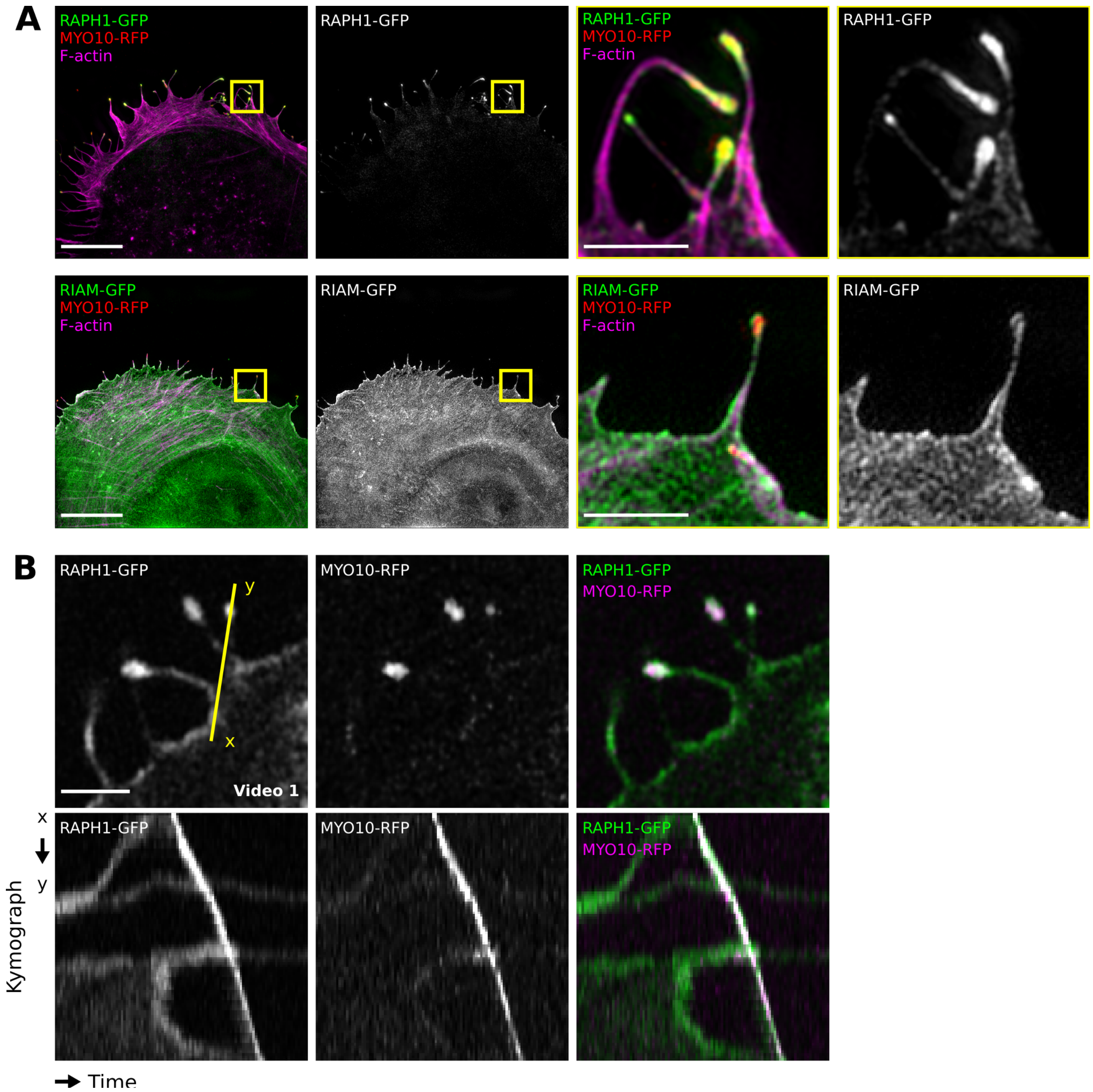


**Fig. S1. RAPH1, but not RIAM, accumulates at the tip of MYO10 filopodia.** (**A**) U2-OS cells expressing MYO10-RFP and RAPH1-GFP or RIAM-GFP were plated on FN for 2 h, fixed, stained for F-actin, and imaged using structured illumination microscopy (SIM). Representative maximum intensity projections (MIPs) are displayed. Scale bars: (main) 20 µm; (inset) 2 µm. (**B**) U2-OS cells expressing MYO10-RFP and RAPH1-GFP were imaged live at high spatiotemporal resolution using an Airyscan confocal microscope. A single time point (upper panel, scale bar: 2 µm) and a kymograph (lower panel) are displayed.

Journal of Cell Science • Supplementary information

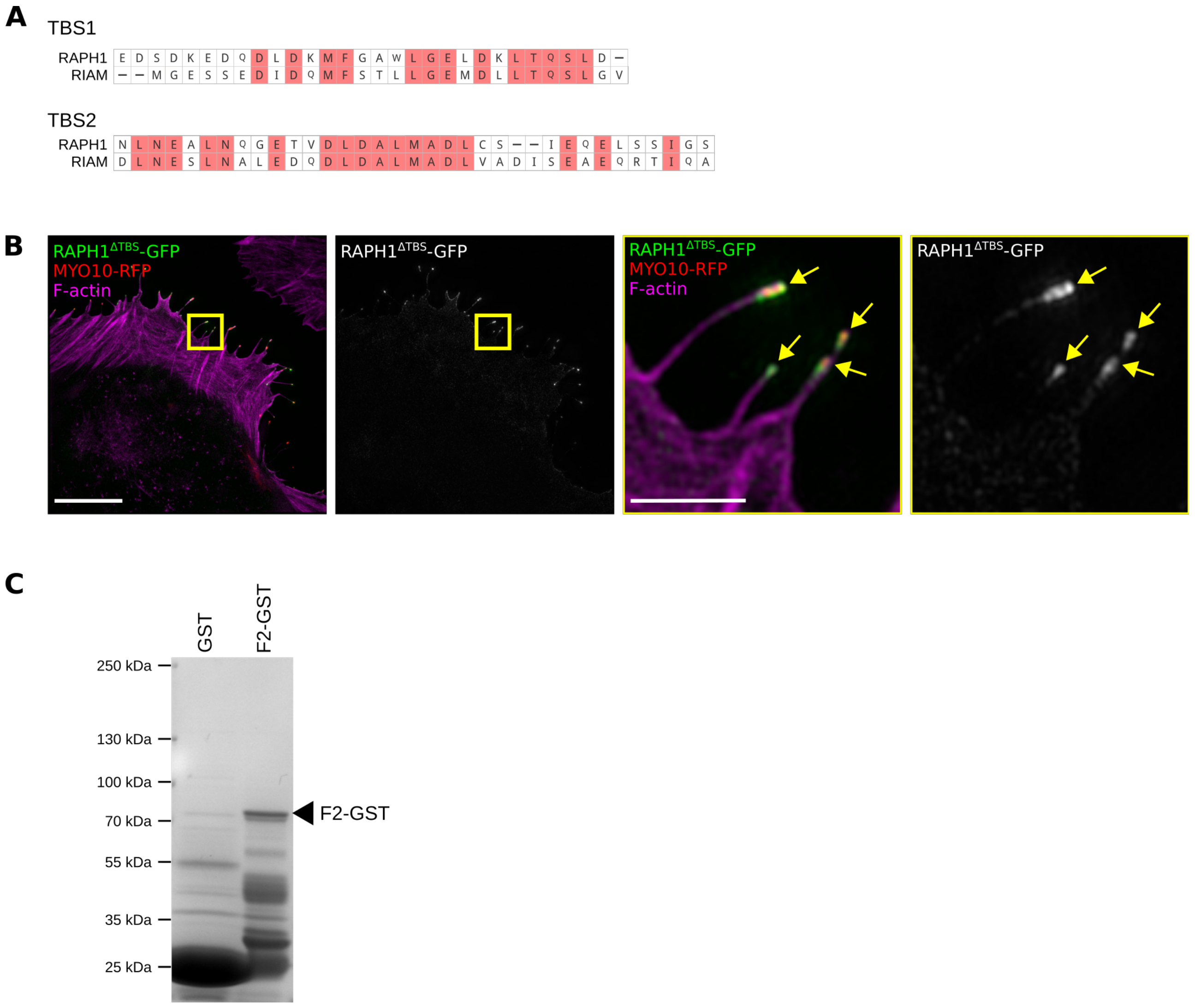


**Fig. S2. RAPH1 putative talin-binding sites do not contribute to its filopodia tip localization.** (**A**) Alignment of the RAPH1 and RIAM talin-binding sites (TBS). (**B**) U2-OS cells expressing MYO10WT-RFP and GFP-RAPH1 lacking both TBS (GFP-RAPH1$^{\Delta TBS}$) were plated on FN for 2 h, fixed, stained for F-actin, and imaged using SIM. Representative MIPs are displayed. Yellow arrows highlight filopodia tips; scale bars: (main) 10 µm; (inset) 2 µm. (**C**) Recombinant GST-RAPH1$^{F2}$ and GST were produced in bacteria and subsequently purified using Glutathione agarose beads. Produced proteins were run on a polyacrylamide gel and stained using coomassie.

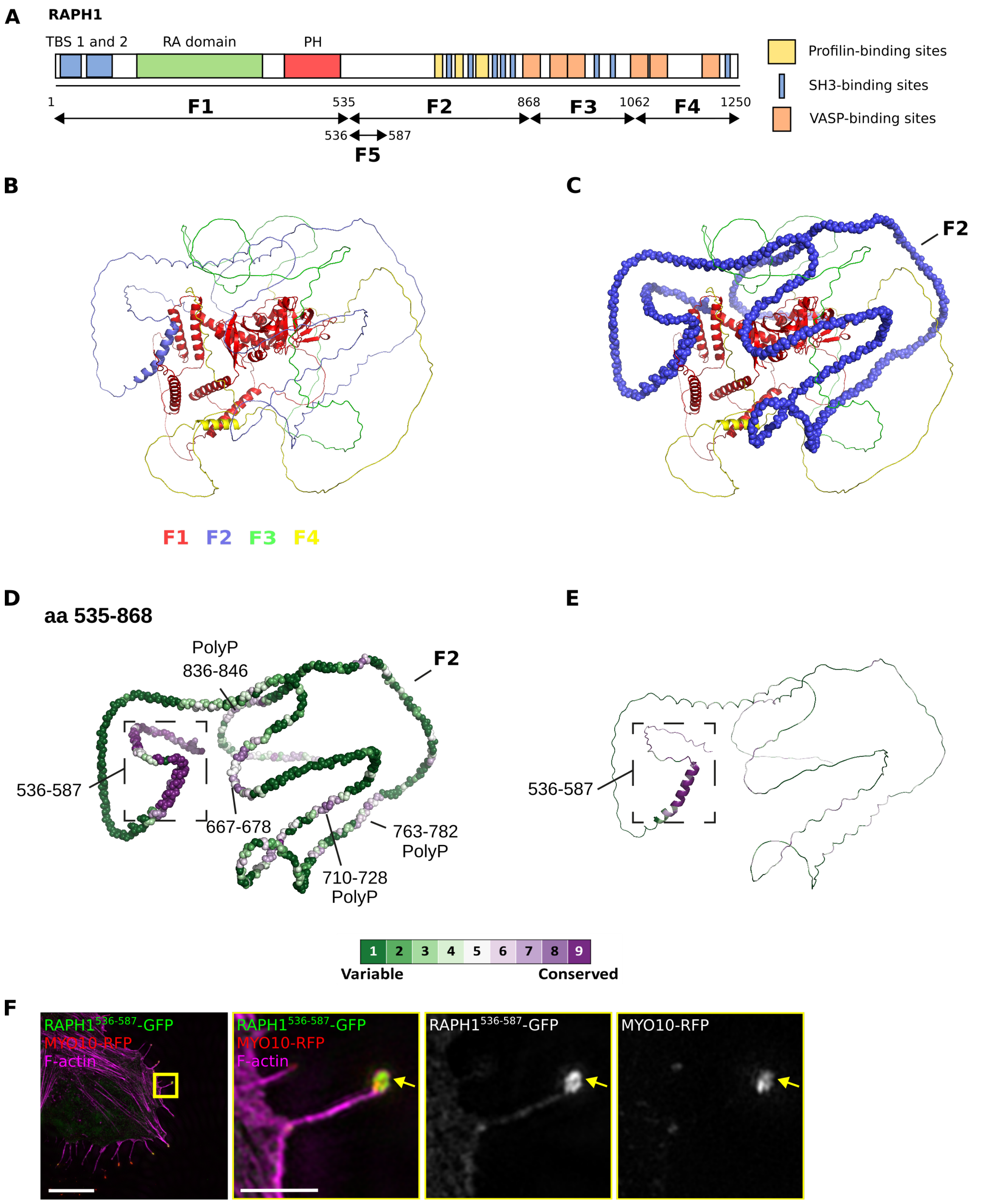


Journal of Cell Science • Supplementary information

**Fig. S3. The MYO10 binding site in RAPH1 is highly conserved.** (**A**) Cartoon of RAPH1 domain structure highlighting the four truncated RAPH1 constructs, F1 to F5. (**B**) Structural model of RAPH1 generated using AlphaFold (Jumper et al., 2021; Varadi et al., 2022) and colored as in (**A**). The majority of RAPH1 protein is predicted to be unstructured. (**C**) The F2 region of RAPH1 was shown to harbor the MYO10 binding site. (**D**-**E**) The F2 region of RAPH1 is colored based on the evolutionary conservation of RAPH1 as determined using the ConSurf server (Ashkenazy et al., 2016). The main chain is shown as spheres (**D**) and cartoons (**E**). Highly conserved regions are shown in purple. The region 536-587 is highly conserved. The poly-proline motifs are also identified using this approach and are highlighted. **(E)** U2-OS cells expressing MYO10WT-RFP and GFP-RAPH1 536-587 were plated on FN for 2 h, fixed, stained for F-actin, and imaged using SIM. Representative MIPs are displayed. Yellow arrows highlight filopodia tips; scale bars: (main) 10 μm; (inset) 2 μm.

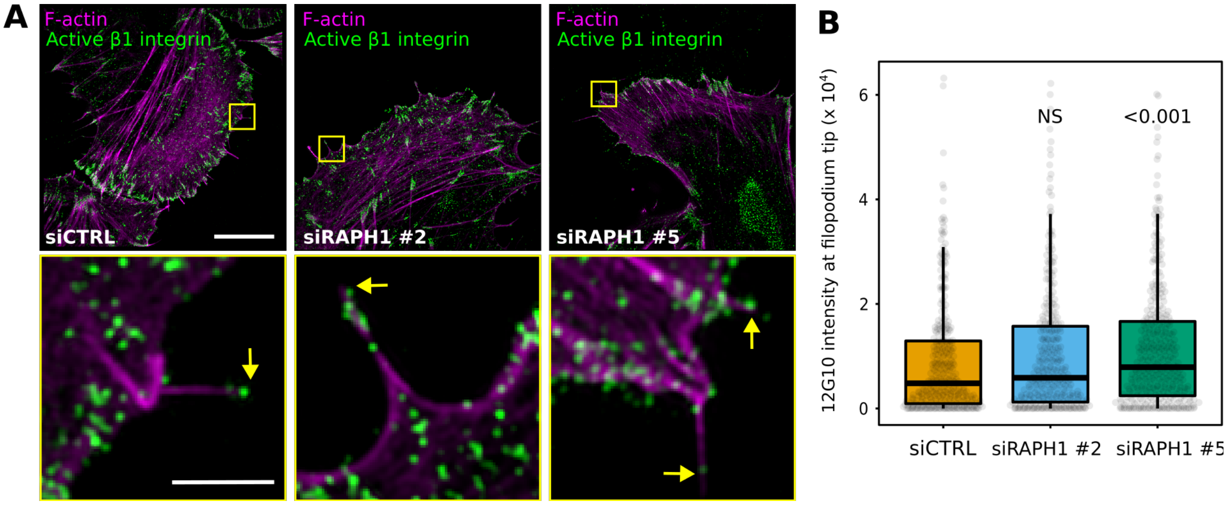


**Fig. S4. RAPH1 is not required for β1-integrin activation at filopodia tips.** (**A**-**B**) RAPH1-silenced U2-OS cells expressing MYO10-GFP were plated on FN for 2 h, stained for active β1-integrin (12G10), and imaged using SIM. Representative MIPs are displayed; scale bars: (main) 20 µm; (inset) 2 µm. (**B**) The average intensity of 12G10 at filopodia tips was measured from line intensity profiles and displayed as boxplots (n > 400 filopodia, three biological repeats). P-values were determined using a randomization test. NS indicates no statistical difference between the mean values of the highlighted condition and the control.

**Table S1.** This file contains the MS analysis of GFP-MYO10FERM and GFP-TalinFERM binding proteins and the MS analysis of biotinylated proteins in cells expressing GFP-MYO10-BioID or GFP-MYO10.

Click here to download Table S1

**Table S2.** This file contains the raw numerical values of the datasets used in this study and their statistical analyses.

Click here to download Table S2

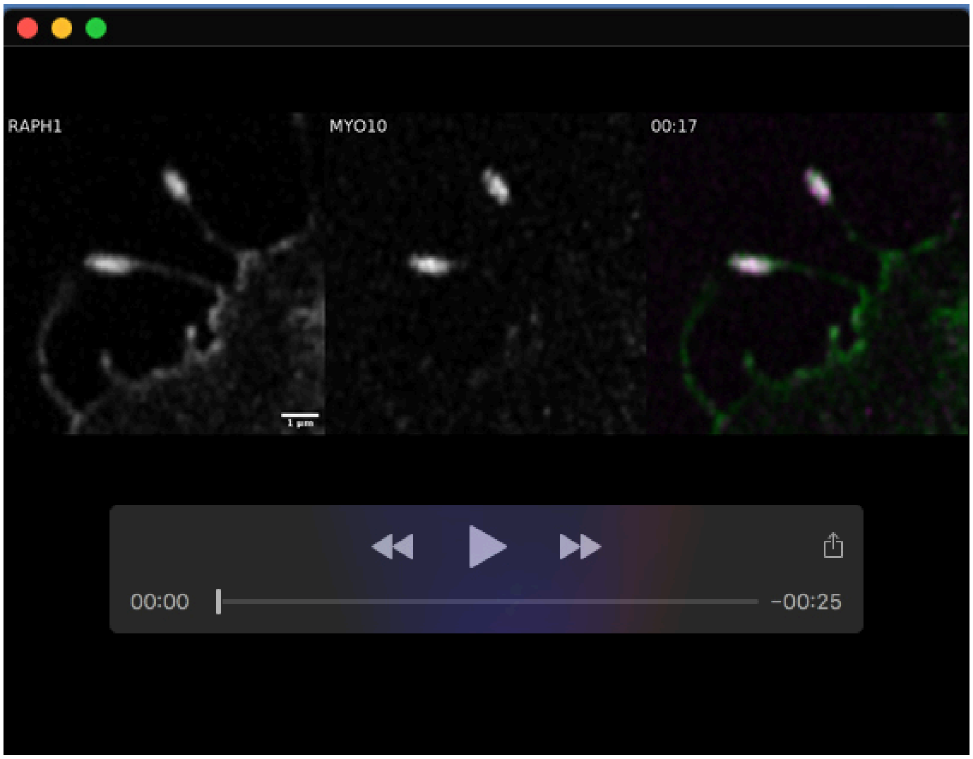


**Movie 1.** U2-OS cells expressing MYO10-RFP and RAPH1-GFP were imaged live at high spatiotemporal resolution using an Airyscan confocal microscope.
