# Supplementary material for: Myosin-X recruits lamellipodin to filopodia tips
Source: J Cell Sci. 2023 Mar 2;136(5):jcs260574. doi: 10.1242/jcs.260574 (PMC10022686; doi:10.1242/jcs.260574)
Supplement: Supplementary file 1 [file joces-136-260574_review_history.pdf]

Myosin-X recruits lamellipodin to filopodia tips

Ana Popović, Mitro Miihkinen, Sujan Ghimire, Rafael Saup, Max L. B. Grönloh, Neil J. Ball, Benjamin T. Goult, Johanna Ivaska and Guillaume Jacquemet
DOI: 10.1242/jcs.260574

Editor: Anne Straube

Review timeline

Original submission:	25 August 2022
Editorial decision:	26 September 2022
First revision received:	25 November 2022
Editorial decision:	10 January 2023
Second revision received:	10 January 2023
Accepted:	12 January 2023

Original submission

First decision letter

MS ID#: JOCES/2022/260574

MS TITLE: Myosin-X recruits lamellipodin to filopodia tips.

AUTHORS: Mitro Miihkinen, Ana Popović, Sujan Ghimire, Rafael Saup, Max L.B. Grönloh, Neil J. Ball, Ben Thomas Goult, Johanna Ivaska, and Guillaume Jacquemet
ARTICLE TYPE: Short Report

We have now reached a decision on the above manuscript.

To see the reviewers' reports and a copy of this decision letter, please go to: https://submit-jcs.biologists.org and click on the 'Manuscripts with Decisions' queue in the Author Area. (Corresponding author only has access to reviews.)

As you will see, the reviewers gave favourable reports but raised some critical points that will require amendments to your manuscript. I hope that you will be able to carry these out because I would like to be able to accept your paper, depending on further comments from reviewers.

Please ensure that you clearly highlight all changes made in the revised manuscript. Please avoid using 'Tracked changes' in Word files as these are lost in PDF conversion.

I should be grateful if you would also provide a point-by-point response detailing how you have dealt with the points raised by the reviewers in the 'Response to Reviewers' box. Please attend to all of the reviewers' comments. If you do not agree with any of their criticisms or suggestions please explain clearly why this is so.

Reviewer 1

Advance summary and potential significance to field

Miihkinen et al describes the identification of lamellipodin as a cargo of the myosin, MyoX. Using a combination of BioID and mass spec after GFP-trap, and biochemical interaction assays the authors identify lamellipodin as a binding partner of the FERM domain of MyoX. They then map a region

© 2023. Published by The Company of Biologists under the terms of the Creative Commons Attribution License (https://creativecommons.org/licenses/by/4.0/).

directly after the RA-PH domains within lamellipodin as the direct binding site for the FERM domain of MyoX. Using siRNA-based knockdown, the authors describe a requirement of lamellipodin for MyoX induced filopodia formation and elongation but not for integrin activation at filopodia tips.

Comments for the author

It was a pleasure to read as this was a well conducted and controlled study with appropriate repetition of experiments. My biggest concern is that the authors did not provide any evidence that lamellipodin is recruited via the F2 region in lamellipodin to MyoX to the tips of filopodia. The mapped binding region of the MyoX FERM domain the F2 region in lamellipodin is around 330 aa. For the authors to claim that lamellipodin is recruited by MyoX to the tips of filopodia, the authors should identify the exact binding site within the F2 region and then mutate full length lamellipodin. Only re-expression of such a mutant lamellipodin cDNA in the lamellipodin knockdown cells can establish whether lamellipodin is solely recruited by this interaction to the tips of filipodia and whether this interaction mediates MyoX function via lamellipodin (Figure 4).

In addition, the authors should verify their knockdown phenotypes in Figure 4 by re-expressing wild-type lamellipodin to control for off-target effects.

The authors need to add the statistical method used and the actual p value for each panel into the figure legend for each figure. Only then the reader can judge whether the correct statistical test was used.

The authors should discuss their findings in comparison to findings from a recent manuscript in BioRxiv (Pokrant et al., BioRxiv; 10.1101/2022.05.12.491613) on MyoX and lamellipodin function in microspikes.

I am happy to support publication in JCS after my concerns have been addressed.

Minor comments:

Page 1: “1-5 mm in length” should read 1-5um.

<u>Reviewer 2</u>

Advance summary and potential significance to field

Myosin-X (MYO10) is an unconventional motor protein that accumulates at the tips of filopodia and drives their formation in various cell types. The present work by Miihkinen and colleagues is an elegant and interesting study in which the authors sought to identify novel Myo10-binding proteins. Using GFP-trap pulldowns and proximity ligation combined with mass spectrometry, they identify RAPH1 (Lamellipodin) as a new interaction partner of MYO10 in U2OS cells even though the negative control in the BioID experiments is not appropriate (see below). The MYO10 MyTH/FERM cargo domain is further shown to interact with the proline/SH3-domain of RAPH1 (referred to as F2), which is quite unexpected, given that other RAPH1 interactors such as VASP and integrins bind to the C-terminus of RAPH1 (F3 and F4). Notably, loss of MYO10-FERM (MYO10-F) results in the inability to recruit RAPH1 to filopodia tips. Moreover, cells transfected with this construct formed fewer and shorter filopodia although these experiments have been conducted with wild type cells (see below). Other than that, and given that the progress made in this work provides important insights into the mechanisms of filopodium formation, I have relatively little to criticize and support publication of this work in JCS after the critical issues have been adequately addressed.

Comments for the author

Specific points:

Fig. 1. BioID experiments: Unfortunately, the authors used GFP-MYO10 as negative control. However, this not an appropriate negative control to exclude unspecific effects in these experiments. Incorporation of a BioID-only control that expresses the biotin ligase without a fused

© 2023. Published by The Company of Biologists under the terms of the Creative Commons Attribution License (https://creativecommons.org/licenses/by/4.0/).

protein of interest has become routine practice to help interpret the list of proteins identified by mass spectrometry in a BioID experiment. The BioID-only control identifies proteins that may be biotinylated randomly due to sheer abundance and/or due to an unnatural affinity to the BioID ligase itself (Roux et al, 2019, Curr Protoc Protein Sci.). For this reason, the BioID experiments must be repeated along with a BioID-only control to obtain information on background biotinylation, and hits then be presented, or at least stated in the text, as x-fold enrichment of prey relative to the BioID-only control.

Fig. 2A. Given that F-actin is shown in magenta, RFP-MYO10, displayed in red, is barely visible. Please add the MYO10-RFP channel separately, as done for RAPH1 and VASP. This also applies to Fig. 2E.

It is not clear to the referee as to why U2OS wild-type but not MYO10-depleted cells were used in these experiments to clearly assess that RAPH1 is recruited to filopodia tips in an MYO10-FERM-dependent manner. MYO10, at least in its active form, is known to form dimers so that MYO10F could heterodimerize with endogenous FL MYO10, thus complicating interpretation of these results. The relative expression levels of GFP-tagged constructs vs endogenous MYO10 needs to be shown. The authors should consider repeating these experiments with MYO10-depleted cells to eliminate impact of endogenous MYO10.

Fig. 4. Please provide enlarged insets showing filopodia in Fig. 4B, as details are barely visible in the current images.
In Fig. 4C showing distribution of filopodium length, the differences appear statistically significant although they are very small. Given that the authors have analyzed 530 filopodia per condition, they might run the risk to reveal statistical difference simply by oversampling. Are the differences still statistically different if data are expressed as average length of filopodia per cell?

As the difference between the data sets in Fig. 4E, depicting MYO10 spot speed and traveled distance, appears to be even smaller, oversampling seems even more likely as the authors analyzed here 9600 filopodia per condition. Are the differences here still statistically different when the data are expressed as average spot speed and traveled distance in filopodia per cell?

In general, mean values of measured parameters (± SD or SEM) are not given anywhere in the text or the figures. See Fig. 2 C, D, F. Fig. 3C and E. Fig. 4B-E. This would be certainly helpful to better compare and assess the differences between respective datasets.

<u>Reviewer 3</u>

Advance summary and potential significance to field

A very nice study showing that RAPH1/lamellipodin is delivered to filopodia tips by Myo10 and that this promotes filopodia formation. The sites in the two proteins that mediate their interaction are identified and the relationship between this complex and VASP localization/function is clarified (although I was surprised a bit that the complex does not also mediate VASP localization to the filopodial tip). These results will definitely be of interest to the JCS readership.

Comments for the author

The data is convincing, the figures are clear, and the writing is excellent-direct and to the point. I only had one tiny request. The sentence "Next, to narrow the list of putative MYO10 cargo, we tagged GFP-MYO10 with the promiscuous biotin ligase BioID (Roux et al., 2012) adjacent to the MYO10 FERM domain." confused me (especially the last part). I know I could wade through the Methods but it would be nice to say more clearly in the text what this construct is. Overall a very nice piece of work.

© 2023. Published by The Company of Biologists under the terms of the Creative Commons Attribution License (https://creativecommons.org/licenses/by/4.0/).

First revision

Author response to reviewers' comments

Reviewer 1 Advance Summary and Potential Significance to Field:
Miihkinen et al describes the identification of lamellipodin as a cargo of the myosin, MyoX. Using a combination of BioID and mass spec after GFP-trap, and biochemical interaction assays the authors identify lamellipodin as a binding partner of the FERM domain of MyoX. They then map a region directly after the RA- PH domains within lamellipodin as the direct binding site for the FERM domain of MyoX. Using siRNA-based knockdown, the authors describe a requirement of lamellipodin for MyoX induced filopodia formation and elongation but not for integrin activation at filopodia tips.

Reviewer 1 Comments for the Author:
It was a pleasure to read as this was a well conducted and controlled study with appropriate repetition of experiments.

We thank the reviewer for a constructive review of our manuscript.

My biggest concern is that the authors did not provide any evidence that lamellipodin is recruited via the F2 region in lamellipodin to MyoX to the tips of filopodia. The mapped binding region of the MyoX FERM domain, the F2 region in lamellipodin is around 330 aa. For the authors to claim that lamellipodin is recruited by MyoX to the tips of filopodia, the authors should identify the exact binding site within the F2 region and then mutate full length lamellipodin. Only re-expression of such a mutant lamellipodin cDNA in the lamellipodin knockdown cells can establish whether lamellipodin is solely recruited by this interaction to the tips of filopodia and whether this interaction mediates MyoX function via lamellipodin (Figure 4).

We thank the reviewer for bringing this forward. Using bioinformatic analyses, we found that, within the RAPH1 F2 region, the amino-acid sequence 536-587 is very well conserved (see new figure S3A-E, and below). Structure predictions indicate that this sequence 536-587 forms a helix reminiscent of the helix found on other proteins, such as DCC, interacting with MYO10 FERM.

Expression of a GFP-RAPH1$^{536\text{-}587}$ construct showed that this construct could localize at the tip of MYO10-positive filopodia (Fig S3F and below). Notably, the deletion of the amino acid sequence 536 to 587 in full-length RAPH1 was sufficient to block MYO10 recruitment to RAPH1 and RAPH1 localization and accumulation at filopodia tips (see new figure 3 and below). Furthermore, we found that silencing of RAPH1 decreases MYO10 filopodia formation, and re-expression of RAPH1$^{\Delta 536\text{-}587}$-GFP in RAPH1 silenced cells could not rescue filopodia formation to the same extent as full-length RAPH1 (see new figure 4C and below). Together these data indicate that the 536-587 region is key for RAPH1 recruitment to filopodia tips. We could not identify the exact amino acid(s) required for the interaction between MYO10 and RAPH1 during the time allocated for the revisions.

© 2023. Published by The Company of Biologists under the terms of the Creative Commons Attribution License (https://creativecommons.org/licenses/by/4.0/).

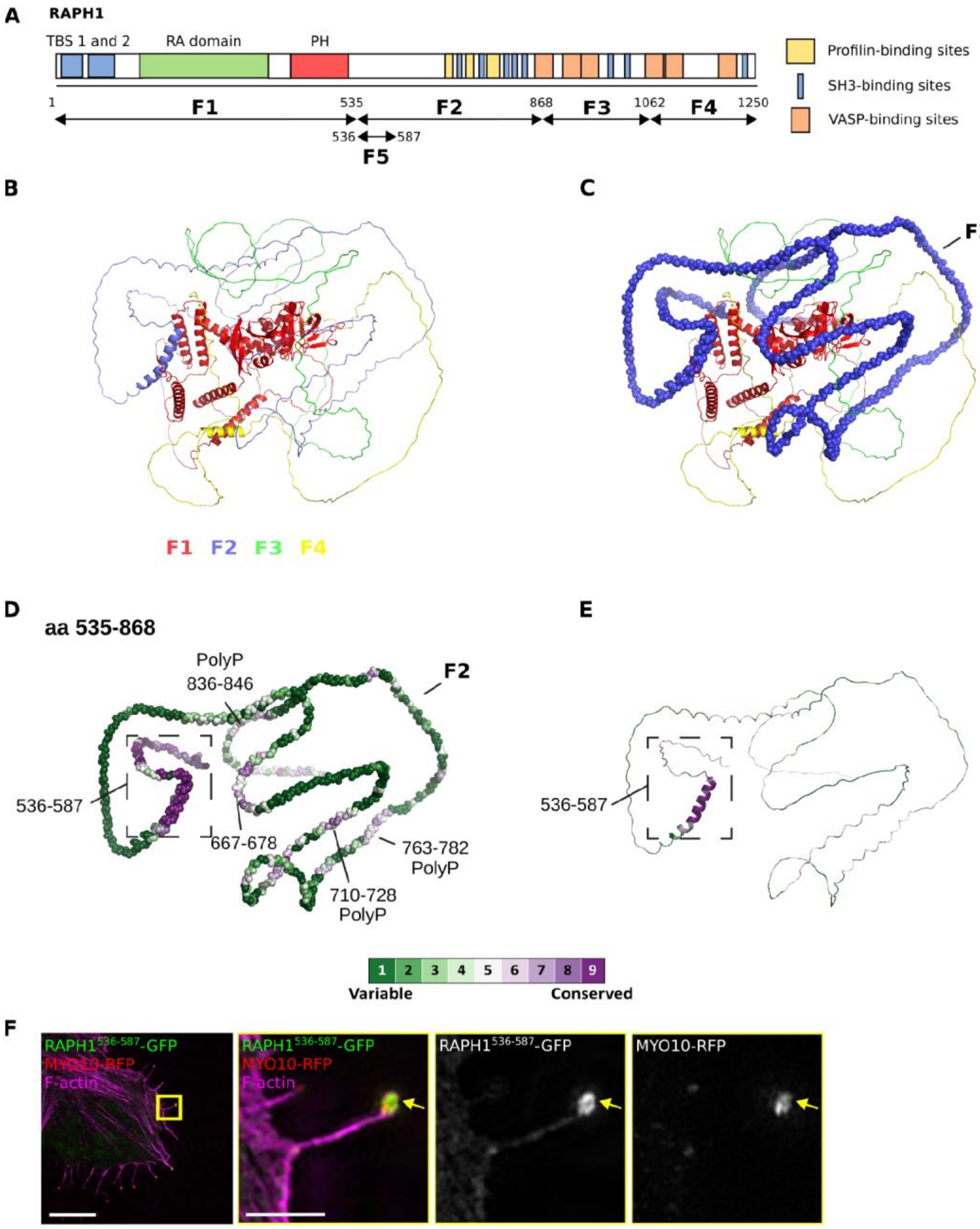

(A) Cartoon of RAPH1 domain structure highlighting the four truncated RAPH1 constructs, F1 to F5. (**B**) Structural model of RAPH1 generated using AlphaFold (Jumper et al., 2021; Varadi et al., 2022) and colored as in (**A**). The majority of RAPH1 protein is predicted to be unstructured. (**C**) The F2 region of RAPH1 was shown to harbor the MYO10 binding site. (**D-E**) The F2 region of RAPH1 is colored based on the evolutionary conservation of RAPH1 as determined using the ConSurf server (Ashkenazy et al., 2016). The main chain is shown as spheres (D) and cartoons (**E**). Highly conserved regions are shown in purple. The region 536-587 is highly conserved. The poly-proline motifs are also identified using this approach and are highlighted.

© 2023. Published by The Company of Biologists under the terms of the Creative Commons Attribution License (https://creativecommons.org/licenses/by/4.0/).

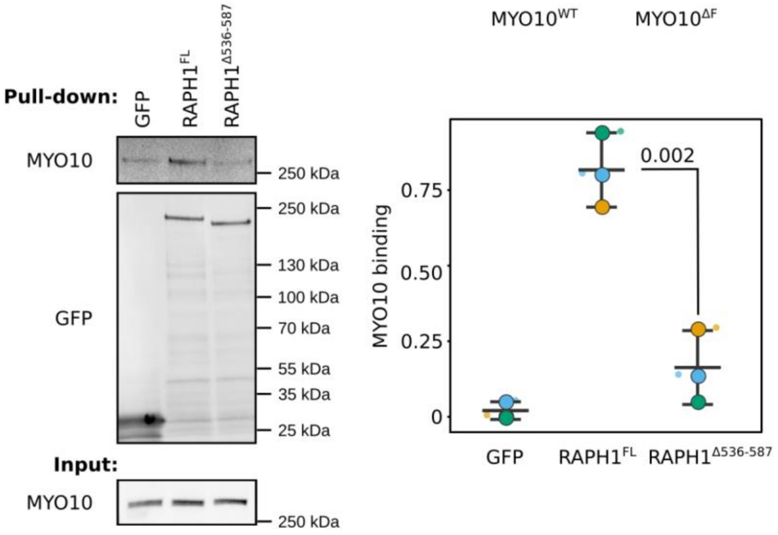

Figure 3 (I) GFP pull-downs in MDA-MB-231 cells expressing full-length RAPH1-GFP, RAPH1-GFP lacking the F5 fragment (RAPH1$^{\Delta 536\text{-}587}$), or GFP alone. Endogenous MYO10 recruitment to the bait proteins was then assessed using a western blot (representative of three biological repeats) (Goedhart, 2021; Lord et al., 2020). The quantifications of MYO10 recruitment to the bait protein are displayed as a SuperPlot where individual repeats are color-coded (p-value calculated using a Welch's t-test).

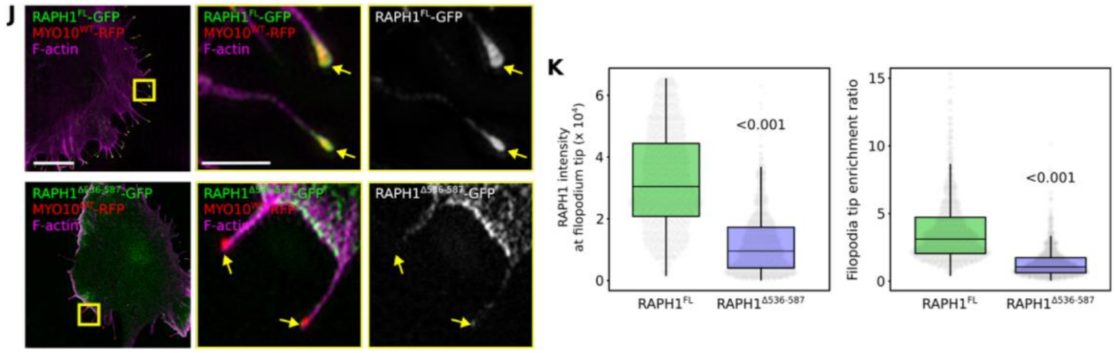

Figure 3 (**J-K**) U2OS cells expressing MYO10-RFP together with full-length RAPH1-GFP (**J**) or RAPH1-GFP lacking the F5 fragment (RAPH1$^{\Delta 536\text{-}587}$) were plated on FN for 2 h, fixed, stained for F-actin, and imaged using SIM. (**J**) Representative MIPs are displayed. Yellow arrows highlight filopodia tips; scale bars: (main) 20 µm; (inset) 2 µm. (**K**) The average intensity and preferential recruitment of the two RAPH1 constructs to filopodia tips are displayed as box plots (n > 800 filopodia per condition; three biological repeats).

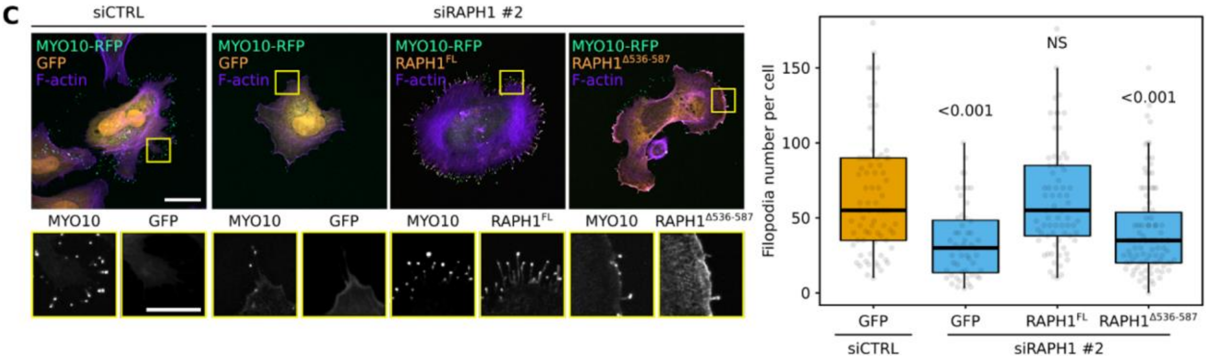

Figure 4. (**C**) RAPH1-silenced U2-OS cells transiently expressing MYO10-RFP together with GFP, RAPH1^{FL}-GFP or RAPH1$^{\Delta 536\text{-}587}$-GFP were plated on FN for 2 h, fixed, and the number of MYO10-positive filopodia per cell was quantified (n > 60 cells per condition, three biological repeats).

© 2023. Published by The Company of Biologists under the terms of the Creative Commons Attribution License (https://creativecommons.org/licenses/by/4.0/).

In addition, the authors should verify their knockdown phenotypes in Figure 4 by re-expressing wild-type lamellipodin to control for off-target effects.

As already mentioned above, we now provide data showing that re-expression of FL-RAPH1 can rescue the filopodia formation in cells previously silenced for RAPH1 (see new figure 4C and below).

C
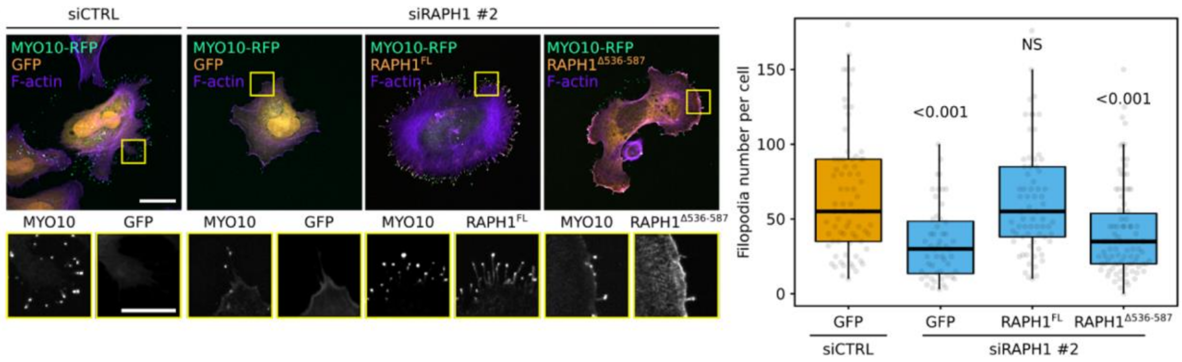

Figure 4. (**C**) RAPH1-silenced U2-OS cells transiently expressing MYO10-RFP together with GFP, RAPH1^{FL}-GFP or RAPH1$^{\Delta536\text{-}587}$-GFP were plated on FN for 2 h, fixed, and the number of MYO10-positive filopodia per cell was quantified (n > 60 cells per condition, three biological repeats)

The authors need to add the statistical method used and the actual p value for each panel into the figure legend for each figure. Only then the reader can judge whether the correct statistical test was used.

We apologize if some information was missing in the previous version of our manuscript. We now ensured that the p-values and the statistical method used to calculate them are indicated in each figure/figure legend. In addition, we directly provide all the raw numerical data used to make the figures and the effect size, which can help appreciate the difference between conditions (Table S2).

The authors should discuss their findings in comparison to findings from a recent manuscript in BioRxiv (Pokrant et al., BioRxiv; 10.1101/2022.05.12.491613) on MyoX and lamellipodin function in microspikes.

We thank the reviewer for bringing this interesting preprint to our attention. Several findings reported by Pokrant et al. support some of our data, and we now highlight and cite this in our revised manuscript.

“Deleting the MYO10 FERM domain led to a loss of RAPH1 accumulation at filopodia tips (Fig. 2A-D), whereas VASP recruitment remained unaffected (Fig. 2A-D). In line with these results, others found that MYO10 FERM was not required for VASP accumulation in microspikes (Pokrant et al., 2022).”

“Next, we investigated the contribution of RAPH1 to filopodia. RAPH1 silencing with two independent siRNA oligos in U2-OS cells expressing MYO10-GFP significantly reduced MYO10-positive filopodia numbers (Fig. 4A and 4B).
Importantly, this phenotype could be rescued by expressing full-length RAPH1 but not by expressing the RAPH1 construct lacking the F5 fragment (Fig. 4C). In line with these results, others found that RAPH1 knock-out reduces MYO10-positive microspike formation in RAT2 cells (Pokrant et al., 2022). “

I am happy to support publication in JCS after my concerns have been addressed.

We thank the reviewer for supporting the publication of our manuscript.

Minor comments:

Page 1: “1-5 mm in length” should read 1-5um.

© 2023. Published by The Company of Biologists under the terms of the Creative Commons Attribution License (https://creativecommons.org/licenses/by/4.0/).

We thank the reviewer for spotting this mistake, which has now been corrected.

Reviewer 2 Advance Summary and Potential Significance to Field:
Myosin-X (MYO10) is an unconventional motor protein that accumulates at the tips of filopodia and drives their formation in various cell types. The present work by Miihkinen and colleagues is an elegant and interesting study in which the authors sought to identify novel Myo10-binding proteins. Using GFP-trap pulldowns and proximity ligation combined with mass spectrometry, they identify RAPH1 (Lamellipodin) as a new interaction partner of MYO10 in U2OS cells even though the negative control in the BioID experiments is not appropriate (see below). The MYO10 MyTH/FERM cargo domain is further shown to interact with the proline/SH3-domain of RAPH1 (referred to as F2), which is quite unexpected, given that other RAPH1 interactors such as VASP and integrins bind to the C-terminus of RAPH1 (F3 and F4). Notably, loss of MYO10-FERM (MYO10-F) results in the inability to recruit RAPH1 to filopodia tips. Moreover, cells transfected with this construct formed fewer and shorter filopodia although these experiments have been conducted with wild type cells (see below). Other than that, and given that the progress made in this work provides important insights into the mechanisms of filopodium formation, I have relatively little to criticize and support publication of this work in JCS after the critical issues have been adequately addressed.

We thank the reviewer for taking the time to carefully assess our manuscript.

Reviewer 2 Comments for the Author:
Specific points:

Fig. 1. BioID experiments: Unfortunately, the authors used GFP-MYO10 as negative control. However, this not an appropriate negative control to exclude unspecific effects in these experiments. Incorporation of a BioID-only control that expresses the biotin ligase without a fused protein of interest has become routine practice to help interpret the list of proteins identified by mass spectrometry in a BioID experiment. The BioID-only control identifies proteins that may be biotinylated randomly due to sheer abundance and/or due to an unnatural affinity to the BioID ligase itself (Roux et al, 2019, Curr Protoc Protein Sci.). For this reason, the BioID experiments must be repeated along with a BioID-only control to obtain information on background biotinylation, and hits then be presented, or at least stated in the text, as x-fold enrichment of prey relative to the BioID-only control.

While we share the reviewer's appreciation for appropriate control, we emphasize that RAPH1 was identified in the study with two different protein pulldown and mass spectrometry approaches. GFP pulldowns using GFP and GFP-Talin FERM as controls and the BioID method. Both approaches specifically identified RAPH1 as a putative MYO10 interactor. Furthermore, RAPH1 interaction with MYO10 was validated extensively with several complementary methods throughout the rest of the manuscript. Thus, repeating all the BioID with the inclusion of a BioID-only control would not provide essential new information to warrant the time and cost required to repeat these mass-spectrometry experiments.

Fig. 2A. Given that F-actin is shown in magenta, RFP-MYO10, displayed in red, is barely visible. Please add the MYO10-RFP channel separately, as done for RAPH1 and VASP. This also applies to Fig. 2E.

We thank the reviewer for suggesting this. We have now updated these figure panels accordingly (see new Figure 2 and below).

© 2023. Published by The Company of Biologists under the terms of the Creative Commons Attribution License (https://creativecommons.org/licenses/by/4.0/).

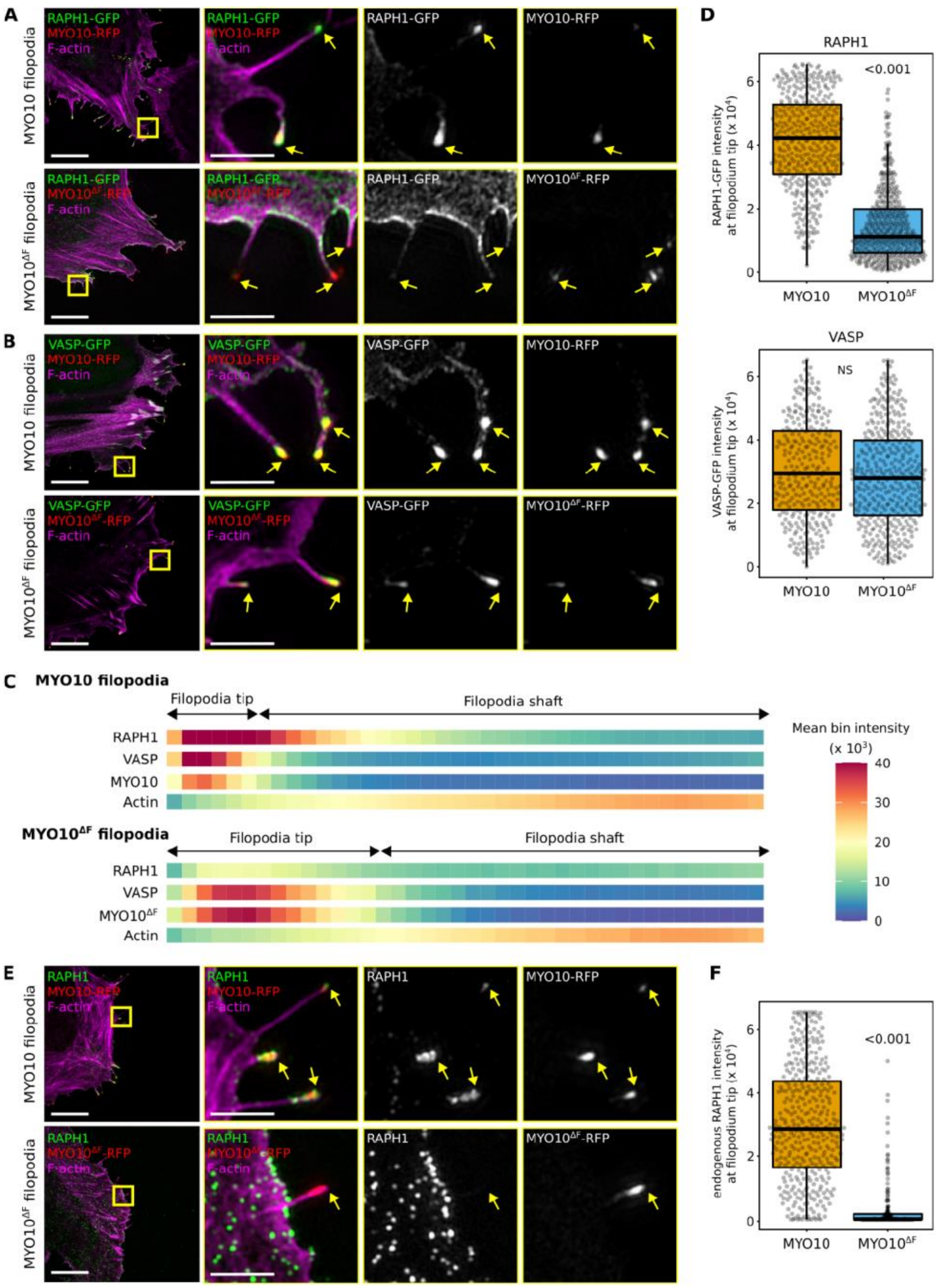

Figure 2: RAPH1 is recruited to filopodia tips in an MYO10-FERM-dependent manner.
(**A-D**) U2OS cells expressing MYO10-RFP or MYO10$^{\Delta F}$-RFP together with RAPH1-GFP (**A**) or VASP-GFP (**B**) were plated on FN for 2 h, fixed, stained for F-actin, and imaged using SIM. (**A-B**) Representative MIPs are displayed. Yellow arrows highlight filopodia tips; scale bars: (main) 20 µm; (inset) 2 µm. (**C**) Heatmap highlighting the sub-filopodial localization of the proteins imaged in (**A** and **B**) generated from intensity profiles (n > 300 filopodia per condition; three biological repeats). (**D**) The average RAPH1 and VASP staining intensity at filopodia tips measured in are displayed as box plots. (**E-F**) U2-OS cells expressing MYO10^{WT}-RFP or MYO10$^{\Delta F}$-RFP were plated on FN for 2 h, fixed, stained for F-actin and endogenous RAPH1, and imaged using SIM. (**E**) A representative ROI is displayed. Yellow arrows highlight filopodia tips; scale bars: 2 µm. (**F**) The average intensity of endogenous RAPH1 at filopodia tips is displayed as box plots (n > 175 filopodia per condition; three biological repeats). For all panels, p-values were determined using a randomization test. NS indicates no statistical difference between the mean values of the highlighted condition and the control.

© 2023. Published by The Company of Biologists under the terms of the Creative Commons Attribution License (https://creativecommons.org/licenses/by/4.0/).

It is not clear to the referee as to why U2OS wild-type but not MYO10-depleted cells were used in these experiments to clearly assess that RAPH1 is recruited to filopodia tips in an MYO10-FERM-dependent manner. MYO10, at least in its active form, is known to form dimers so that MYO10F could heterodimerize with endogenous FL MYO10, thus complicating interpretation of these results. The relative expression levels of GFP-tagged constructs vs endogenous MYO10 needs to be shown. The authors should consider repeating these experiments with MYO10-depleted cells to eliminate impact of endogenous MYO10.

We thank the reviewer for highlighting this important point. Endogenous MYO10 levels are very low in U2OS compared to other cancer cell lines (up to a 12-fold difference, see below). This is why we choose to overexpress MYO10 in this background. In addition, we did not silence endogenous MYO10 when over-expressing MYO10ΔF as we extensively characterized previously that MYO10ΔF acts as a dominant negative in U2OS cells (Miihkinen et al., 2021). We now clearly specify this in the revised manuscript:

“It was not necessary to suppress the expression of endogenous MYO10 here as MYO10ΔF has a dominant-negative effect in U2OS cells (Miihkinen et al., 2021).”

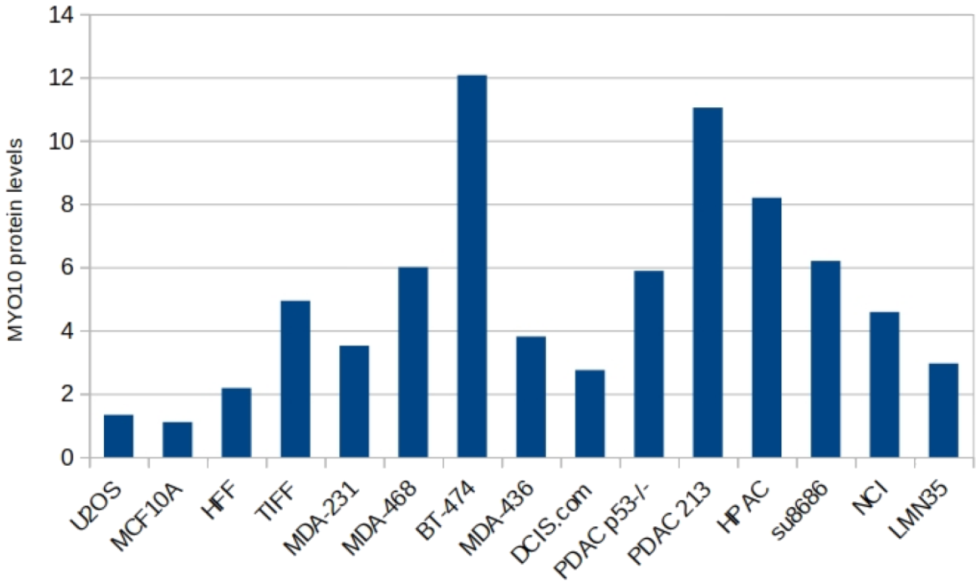

MYO10 protein levels in various cell lines as measured by western blot.

We previously generated and published a stable line that constitutively expresses MYO10-GFP (Jacquemet et al. 2016). We observed a similar number of filopodia using this cell line than when MYO10-GFP or mScarlet is transiently expressed. When MYO10 is stably expressed in U2OS cells, the expression levels are as found below (figures for reviewer only):

NOTE: We have removed data that had been provided for the referees in confidence.

U2OS stable line that constitutively expresses MYO10-GFP (Figure published in Jacquemet et al. 2016).

Fig. 4. Please provide enlarged insets showing filopodia in Fig. 4B, as details are barely visible in the current images.

We thank the reviewer for suggesting this. We have now updated this figure accordingly (see new figure 4B and below).

© 2023. Published by The Company of Biologists under the terms of the Creative Commons Attribution License (https://creativecommons.org/licenses/by/4.0/).

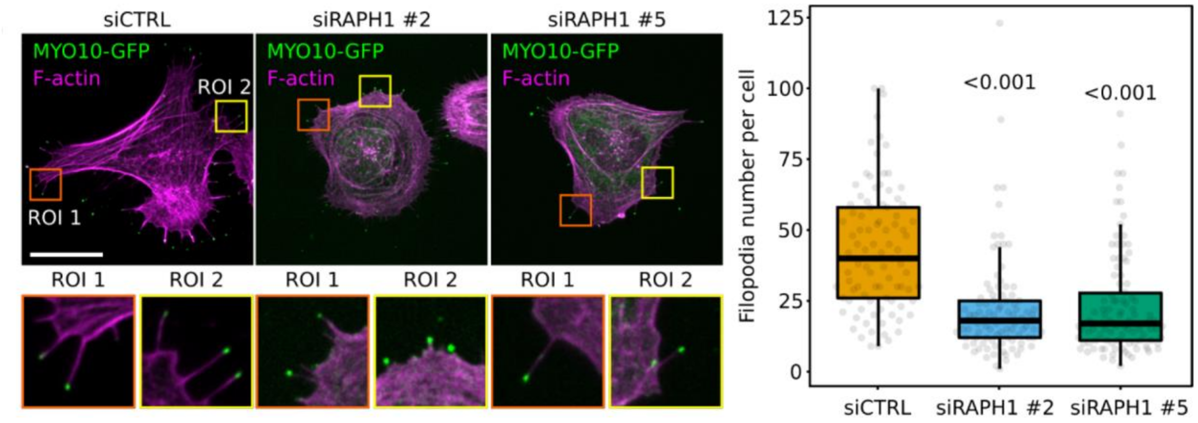

Figure 4B: (B) RAPH1-silenced U2-OS cells transiently expressing MYO10-GFP were plated on FN for 2 h, fixed, and the number of MYO10-positive filopodia per cell was quantified (n > 93 cells per condition, three biological repeats).

In Fig. 4C showing distribution of filopodium length, the differences appear statistically significant although they are very small. Given that the authors have analyzed 530 filopodia per condition, they might run the risk to reveal statistical difference simply by oversampling. Are the differences still statistically different if data are expressed as average length of filopodia per cell? As the difference between the data sets in Fig. 4E, depicting MYO10 spot speed and traveled distance, appears to be even smaller, oversampling seems even more likely as the authors analyzed here 9600 filopodia per condition. Are the differences here still statistically different when the data are expressed as average spot speed and traveled distance in filopodia per cell?

We thank the reviewer for highlighting this point. Here we bring forward that there are better solutions than averaging the data to overcome a possible issue due to oversampling. Averaging the data creates more problems than it solves. Instead, statistical methods less sensitive to sample size, such as the randomization test we use, are more suitable. That said, no statistical method (including the one we use) is entirely independent of sampling size. Therefore, we now directly provide all the raw numerical data used to make the figures and the effect size, which can help the reader appreciate the difference between conditions (Table S2). In contrast with p-values, the effect size is independent of the sample size. We also provide several other useful statistical values (mean, sd, sem, 95CI mean, median, MAD, IQR, 95CI median).

Regarding the filopodia size and the MYO10 spots, speed/ distance traveled datasets, the differences, while small, are real. Importantly, the differences are observed with both lamellipodin siRNAs. However, the critical question, in our opinion, is the biological relevance of these small differences. We now clearly highlight this in the text:

"Finally, we explored the role of RAPH1 in modulating filopodia dynamics in control and RAPH1-silenced U2-OS cells expressing MYO10-GFP. While the overall filopodia lifetime was unaffected after RAPH1 depletion, MYO10 puncta moved slightly faster and over longer distances than in control cells (Fig. 4E and 4F), indicating that the filopodia tip complex is more dynamic in RAPH1-depleted cells. The biological significance of these differences remains, however, to be investigated."

In general, mean values of measured parameters (± SD or SEM) are not given anywhere in the text or the figures. See Fig. 2 C, D, F. Fig. 3C and E. Fig. 4B-E. This would be certainly helpful to better compare and assess the differences between respective datasets.

As described above, we also provide these statistical values in Table S2.

Reviewer 3 Advance Summary and Potential Significance to Field:
A very nice study showing that RAPH1/lamellipodin is delivered to filopodia tips by Myo10 and that this promotes filopodia formation. The sites in the two proteins that mediate their interaction are identified and the relationship between this complex and VASP localization/function is clarified

© 2023. Published by The Company of Biologists under the terms of the Creative Commons Attribution License (https://creativecommons.org/licenses/by/4.0/).

(although I was surprised a bit that the complex does not also mediate VASP localization to the filopodial tip). These results will definitely be of interest to the JCS readership.

Reviewer 3 Comments for the Author:
The data is convincing, the figures are clear, and the writing is excellent- direct and to the point. I only had one tiny request. The sentence "Next, to narrow the list of putative MYO10 cargo, we tagged GFP-MYO10 with the promiscuous biotin ligase BioID (Roux et al., 2012) adjacent to the MYO10 FERM domain." confused me (especially the last part). I know I could wade through the Methods but it would be nice to say more clearly in the text what this construct is. Overall a very nice piece of work.

We thank the reviewer for the positive feedback and for supporting the publication of our manuscript. We have now rephrased the sentence highlighted by the reviewer to:

“Next, to narrow the list of putative MYO10 cargo, we tagged GFP-MYO10 with the promiscuous biotin ligase BioID (Roux et al., 2012). BioID was tagged in C-terminal, just after the MYO10 FERM domain.”

Second decision letter

MS ID#: JOCES/2022/260574

MS TITLE: Myosin-X recruits lamellipodin to filopodia tips.

AUTHORS: Ana Popović, Mitro Miihkinen, Sujan Ghimire, Rafael Saup, Max L.B. Grönloh, Neil J. Ball, Ben Thomas Goult, Johanna Ivaska, and Guillaume Jacquemet
ARTICLE TYPE: Short Report

We have now reached a decision on the above manuscript.

To see the reviewers' reports and a copy of this decision letter, please go to: https://submit-jcs.biologists.org and click on the 'Manuscripts with Decisions' queue in the Author Area. (Corresponding author only has access to reviews.)

As you will see, the reviewers gave favourable reports but reviewer 2 raises some critical points that will require minor amendments to your manuscript. I don't think that the point about the BioID control needs addressing as the findings have been confirmed with different methods, but please address the other points, so that I will be able to accept your paper.

Please ensure that you clearly highlight all changes made in the revised manuscript. Please avoid using 'Tracked changes' in Word files as these are lost in PDF conversion.

I should be grateful if you would also provide a point-by-point response detailing how you have dealt with the points raised by the reviewers in the 'Response to Reviewers' box. Please attend to all of the reviewers' comments. If you do not agree with any of their criticisms or suggestions please explain clearly why this is so.

Reviewer 1

Advance summary and potential significance to field

The authors addressed all my concerns and the revised manuscript is significantly improved. I am now happy to support the publication of this manuscript which reports important, exciting findings.

© 2023. Published by The Company of Biologists under the terms of the Creative Commons Attribution License (https://creativecommons.org/licenses/by/4.0/).

Comments for the author

N/A

Reviewer 2

Advance summary and potential significance to field

The revision of the manuscript by Miihkinen and colleagues is, in my opinion, insufficient.

Comments for the author

The authors state that a BioID control would not provide significant new information to justify the time and expense of repeating these experiments. This argumentation is not acceptable, because a scientific experiment without an appropriate negative control is absolutely useless. Thus, the authors must either repeat the experiments including the BioID-empty negative control in order to demonstrate that RAPH1 is specifically biotinylated by the MYO10-BioID bait, or remove all BioID data from the manuscript.

Reviewer #1 suggested the authors to discuss their results in comparison with the results of a recent manuscript posted in BioRxiv (Pokrant et al. 2022) on the function of MyoX and RAPH1 in microspikes. Unfortunately, this is not well done. In line 122 the authors state “Deleting the MYO10 FERM domain led to a loss of RAPH1 accumulation at filopodia tips (Fig. 2A-D), whereas VASP recruitment remained unaffected (Fig. 2A-D). In line with these results, others found that MYO10 FERM was not required for VASP accumulation in microspikes (Pokrant et al., 2022).” However, there is no mention and discussion of the observation from the Pokrant study that MYO10 lacking the FERM domain also fails to recruit RAPH1 to microspike tips, which seems worthwhile discussing in the context of the present work.

Related to the Pokrant study in line 182 the authors state: “In line with these results, others found that RAPH1 knock-out reduces MYO10-positive microspike formation in RAT2 cells.” This statement seems not be correct. As I understand the paper, MYO10 but not RAPH1 was knocked down in RAT2 cells to show that loss of MYO10 prevents microspike formation. This paragraph needs to be reworded.

The authors emphasize the statistical significance of their data, but the mean values of the measured parameters (± SD or SEM) are still not given anywhere in the text or in the figure. This makes it quite difficult for the reader, as he/she always has to look for these values in Table S2, so that he/she has to jump back and forth all the time.

Reviewer 3

Advance summary and potential significance to field

Please see my first review.

Comments for the author

Apologies for taking so long- A very nice study that should be published as is.

© 2023. Published by The Company of Biologists under the terms of the Creative Commons Attribution License (https://creativecommons.org/licenses/by/4.0/).

Second revision

Author response to reviewers' comments

Reviewer 1 Advance Summary and Potential Significance to Field:
The authors addressed all my concerns and the revised manuscript is significantly improved. I am now happy to support the publication of this manuscript which reports important, exciting findings.

Reviewer 1 Comments for the Author:
N/A

We thank the reviewer for recommending the publication of our manuscript.

Reviewer 2 Advance Summary and Potential Significance to Field:
The revision of the manuscript by Miihkinen and colleagues is, in my opinion, insufficient.

We thank the reviewer for taking the time to evaluate our manuscript further.

Reviewer 2 Comments for the Author:
The authors state that a BioID control would not provide significant new information to justify the time and expense of repeating these experiments. This argumentation is not acceptable, because a scientific experiment without an appropriate negative control is absolutely useless. Thus, the authors must either repeat the experiments including the BioID-empty negative control in order to demonstrate that RAPH1 is specifically biotinylated by the MYO10-BioID bait, or remove all BioID data from the manuscript.

Here we would like to respectfully disagree with the reviewer. Our BioID experiments already contain an appropriate control which is to identify the protein biotinylated present in cells when they are stimulated by biotin (background). Our mass-spectrometry and western blot experiments clearly show that RAPH1 is specifically biotinylated in cells as a result of MYO10-BioID expression. In this context, identifying the proteins biotinylated in cells over-expressing cytoplasmic BioID (localizing everywhere) would be largely irrelevant. Using our BioID data, we speculated that MYO10 could interact (directly) with RAPH1, a result that we extensively cross-validated throughout the manuscript.

Reviewer #1 suggested the authors to discuss their results in comparison with the results of a recent manuscript posted in BioRxiv (Pokrant et al. 2022) on the function of MyoX and RAPH1 in microspikes. Unfortunately, this is not well done. In line 122 the authors state “Deleting the MYO10 FERM domain led to a loss of RAPH1 accumulation at filopodia tips (Fig. 2A-D), whereas VASP recruitment remained unaffected (Fig. 2A-D). In line with these results, others found that MYO10 FERM was not required for VASP accumulation in microspikes (Pokrant et al., 2022).” However, there is no mention and discussion of the observation from the Pokrant study that MYO10 lacking the FERM domain also fails to recruit RAPH1 to microspike tips, which seems worthwhile discussing in the context of the present work.

We thank the reviewer for highlighting this very exciting piece of data that was unfortunately buried in the supplementary figures of Pokrant et al. 2022. We apologize for missing it. This is now rectified in our manuscript. “ In line with our results, others reported that MYO10 FERM was required for RAPH1 but not for VASP accumulation in microspikes (Pokrant et al., 2023).”

Related to the Pokrant study in line 182 the authors state: “In line with these results, others found that RAPH1 knock-out reduces MYO10-positive microspike formation in RAT2 cells.” This statement seems not be correct. As I understand the paper, MYO10 but not RAPH1 was knocked down in RAT2 cells to show that loss of MYO10 prevents microspike formation. This paragraph needs to be reworded.

We thank the reviewer for noticing this mistake. Indeed, Pokrant et al. used B16-F1 cells for these experiments (Pokrant et al Fig. 2I). This has now been corrected in the manuscript. We have now rephrased the text to:

© 2023. Published by The Company of Biologists under the terms of the Creative Commons Attribution License (https://creativecommons.org/licenses/by/4.0/).

"In line with these results, others found that RAPH1 knock-out reduces microspike formation in B16-F1 cells (Pokrant et al., 2023)."

The authors emphasize the statistical significance of their data, but the mean values of the measured parameters (± SD or SEM) are still not given anywhere in the text or in the figure. This makes it quite difficult for the reader, as he/she always has to look for these values in Table S2, so that he/she has to jump back and forth all the time.

Here we would like to respectfully disagree with the reviewer. We find that adding the mean values of the measured parameters (± SD or SEM) throughout the text strongly disrupts the flow of the text, making it harder to read. In addition, these parameters do not reflect the statistical significance of the data as they only poorly describe the actual raw data distribution. If not required by editorial guidelines, we would prefer to keep these values in Table S2.

Reviewer 3 Advance Summary and Potential Significance to Field:
Please see my first review.

Reviewer 3 Comments for the Author:

Apologies for taking so long- A very nice study that should be published as is.

We thank the reviewer for recommending the publication of our manuscript.

Third decision letter

MS ID#: JOCES/2022/260574

MS TITLE: Myosin-X recruits lamellipodin to filopodia tips.

AUTHORS: Ana Popović, Mitro Miihkinen, Sujan Ghimire, Rafael Saup, Max L.B. Grönloh, Neil J. Ball, Ben Thomas Goult, Johanna Ivaska, and Guillaume Jacquemet
ARTICLE TYPE: Short Report

I am happy to tell you that your manuscript has been accepted for publication in Journal of Cell Science, pending standard ethics checks.

© 2023. Published by The Company of Biologists under the terms of the Creative Commons Attribution License (https://creativecommons.org/licenses/by/4.0/).
